# Phase Transition Driven Zn‐Ion Battery With Laser‐Processed V_2_C/V_2_O_5_ Electrodes for Wearable Temperature Monitoring

**DOI:** 10.1002/smll.202409987

**Published:** 2024-12-29

**Authors:** Sujit Deshmukh, Jayraj V. Vaghasiya, Jan Michalička, Rostislav Langer, Michal Otyepka, Martin Pumera

**Affiliations:** ^1^ Future Energy and Innovation Laboratory Central European Institute of Technology Brno University of Technology Purkyňova 123 Brno 61200 Czech Republic; ^2^ Central European Institute of Technology Brno University of Technology Purkyňova 123 Brno 61200 Czech Republic; ^3^ IT4Innovations VSB‐Technical University Ostrava 17. listopadu 2172/15 Ostrava‐Poruba 708 00 Czech Republic; ^4^ Regional Centre of Advanced Technologies and Materials Czech Advanced Technology and Research Institute (CATRIN) Palacký University in Olomouc Šlechtitelů 27 Olomouc 78371 Czech Republic; ^5^ Faculty of Electrical Engineering and Computer Science VSB – Technical University of Ostrava 17. listopadu 2172/15 Ostrava 70800 Czech Republic; ^6^ Department of Medical Research China Medical University Hospital China Medical University No. 91 Hsueh‐Shih Road Taichung Taiwan

**Keywords:** aqueous zinc ion battery, cyclic stability, temperature sensor, vanadium carbide, vanadium oxide, wearable bioelectronics

## Abstract

Flexible power supply devices present significant potential for wearable bioelectronics within the Internet of Things. Aqueous zinc‐ion batteries have emerged as a viable and safe alternative for power supply in flexible electronics. Nevertheless, typical battery behaviors are generally detrimental with unfavorable phase transition of electrodes, which invariably lead to rapid performance degradation. Here, extraordinary capacity enhancement of 150% is presented, sustained over 60 000 cycles, attained using vanadium carbide MXene (V_2_C)/vanadium pentoxide (V_2_O_5_) heterostructure as cathode. The unique cathode material is created through the rational engineering of MAX (V_2_AlC), employing a single‐step laser writing process. The ultrastable Zn ion battery stands in stark contrast to all previously reported counterparts, which typically exhibit capacity degradation within a few hundred/thousand cycles. The primary mechanisms driving this enhancement include the delamination of V_2_C MXene and an unexpected favorable phase transition during cycling. Additionally, a wearable power supply is constructed using a series configuration and is integrated with a commercial temperature sensor for wireless, real‐time body temperature monitoring. This study highlights the critical role of electrode design for advanced wearable bioelectronics.

## Graphical abstract

Wearable bioelectronics demands flexible and durable power sources. This study introduces an ultrastable aqueous Zn‐ion battery with a 150% capacity enhancement over 60 000 cycles, using a vanadium carbide (V₂C)/vanadium pentoxide (V₂O₅) heterostructure cathode engineered via laser processing. The battery powers a wearable temperature sensor for wireless monitoring, emphasizing innovative electrode design for next‐generation Internet of Things applications.

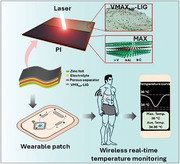

## 1 Introduction

Wearable and flexible electronics, a distinctive field in modern electronics, have showcased versatile applications in customized healthcare sensors,^[^ ^1^, ^2^ ^]^ synthetic electronic skin, implantable medical devices, and actuated soft robots.^[^ ^3^, ^4^, ^5^, ^6^ ^]^ The future commercialization of wearable electronics relies significantly on flexible batteries for energy autonomy.^[^ ^7^, ^8^, ^9^ ^]^ Unlike traditional bulky and heavy batteries, flexible batteries are thin, lightweight, and capable of synchronous actuation without imposing localized constraints.^[^ ^7^ ^]^ This has spurred continuous and intense research interest in developing flexible energy solutions to propel the advancement of wearable technology.^[^ ^10^, ^11^, ^12^ ^]^

Ensuring user safety with flexible batteries is crucial, given that these devices undergo repeated deformations in close contact with the human body. While the current landscape of electrochemical (EC) energy storage is dominated by lithium‐ion batteries,^[^ ^13^ ^]^ their effect on the environment and safety concerns, particularly in the biomedical sector, restrict their application.^[^ ^14^, ^15^, ^16^ ^]^ As a solution, rechargeable aqueous batteries, utilizing water‐based electrolytes, emerge as promising alternatives for flexible bioelectronics.^[^ ^17^ ^]^ They offer good safety, easy assembly, and environmental friendliness, addressing the limitations associated with traditional batteries in the context of flexible devices close to the human body. Specifically, the special qualities of the zinc anode make aqueous zinc ion batteries (AZIBs) function better than other types due to the unique properties of the Zn anode, including its high abundance, widespread availability, low cost, nontoxicity, and high capacity of 820 mAh g^−1^.^[^ ^18^ ^]^

However, creating a cathode material with both high capacity and sustained cyclic performance poses a significant hurdle, mostly because of the strong polarization of bivalent Zn^2+^.^[^ ^19^ ^]^ For example, Prussian blue equivalents offer only restricted capacity (≈50 mAh g^−1^),^[^ ^20^ ^]^ and different phases of MnO_2_ (α, β, γ, δ, and λ) exhibit rapid capacity fading.^[^ ^21^, ^22^, ^23^, ^24^ ^]^ Despite enhancements in the cyclic performance of Zn/MnO_2_ batteries through the introduction of MnSO_4_ in ZnSO_4_ aqueous based electrolyte, the rate capabilities of AZIBs still fall short of meeting practical application expectations.^[^ ^19^ ^]^ During cyclic processes, the interaction between cathode and electrolyte in aqueous environments often triggers structural or phase transitions, negatively impacting cycle life and absolute capacity. Byproducts formed during charge–discharge process are inactive for battery reactions, gradually consuming active cathode materials.^[^ ^25^, ^26^, ^27^ ^]^ Current research emphasizes stable cathode materials or strategies for stabilization. Conversely, if byproducts exhibit more EC activity than the initial cathode materials, the battery may maintain or enhance performance. Achieving high‐performance and ultrastable batteries remains challenging due to the difficulty of imparting multiple functions, such as ion storing capacity, phase evolution, and active secondary byproducts.

The exploration of vanadium‐based cathode materials for zinc‐ion batteries (ZIBs) is driven by vanadium's high natural abundance, multiple valence states and cost‐effectiveness.^[^ ^28^, ^29^ ^]^ Vanadium‐based MXenes, in particular, are emphasized due to their multivalent properties, which make them especially suitable for hosting Zn^2+^ ions.^[^ ^30^ ^]^ For instance, Li et al. demonstrated a phase transition of V_2_CT*_x_* resulting in a gradual capacity increase during cyclic processes.^[^ ^31^ ^]^ Additionally, Liu et al. showed that the valence state of vanadium in V_2_CT*_x_* can be electrochemically activated by varying surface voltages. They achieved optimal EC performance with a cut‐off voltage set at 1.8 V.^[^ ^32^ ^]^ Although V_2_CT*_x_* demonstrates excellent EC performance, the impact of cut‐off voltage and phase evolution on its mechanism is not well understood. Additionally, the use of strong acids (such as HCl or HF) in its synthesis poses safety risks, limiting practical applications.

The vanadium‐based MAX material possesses higher corrosion resistance and it is used mainly as a precursor for MXenes production through acid (HCl or HF) etching. Only a few works have been reported on the usability of the MAX phase.^[^ ^33^ ^]^ Herein we reported chemical‐free Nd‐YAG laser process of the MAX (V_2_AlC) phase to prepare MXene (V_2_C) nanoparticles (NPs) that are linked with current collector graphene network through V–O–C covalent bonding. These particles are below 30 nm and exhibit noteworthy properties due to the electrons confinement within a small volume of nanometric dimension. The in situ MXene functionalized graphene continuously exposes active sites, and the phase transition byproducts consistently contribute to capacity, leading to unusual enhancement of capacity. This distinctive phenomenon, which has not been seen in MXene oxidation derivatives despite thorough EC investigations, leads to an exceptionally long cycle life of up to 60 000 cycles, surpassing all state of art MXene‐based ZIBs (aqueous/organic) in terms of stability. Furthermore, leveraging its flexibility and lightweight properties, our ZIB battery was integrated with a thermal sensor patch to enable real‐time monitoring of human body temperature via a mobile application.

## 2 Results and Discussions

A single‐step laser processing (pulsed Nd‐YAG laser, 532 nm) method was employed to prepare vanadium‐based NPs decorated graphene, with detailed synthesis procedures outlined in the experimental section. We utilize a simple lasing technique on a V_2_AlC‐coated flexible polyimide sheet (PIS) using a Nd:YAG laser (diode‐pumped solid pulsed beam). The energy delivered by an Nd‐YAG pulsed laser beam, focused on a nanoscale area in a brief nanosecond pulse, imparts substantial power to a small particle volume. This intense energy over a short duration induces significant thermal and mechanical stress, resulting in the fragmentation of V_2_AlC sheets into small, spherical MXene particles. Simultaneously, the PIS absorbs infrared energy, producing temperatures over 2000 K in less than a millisecond, which facilitates the photothermal conversion of the PIS into graphene. This approach offers several advantages over existing methods: it eliminates the need for hazardous concentrated HF and effectively utilizes the MAX phase, leading to the in situ bond formation between MXene particles and the graphene network in a single step.

**Scheme 1:**
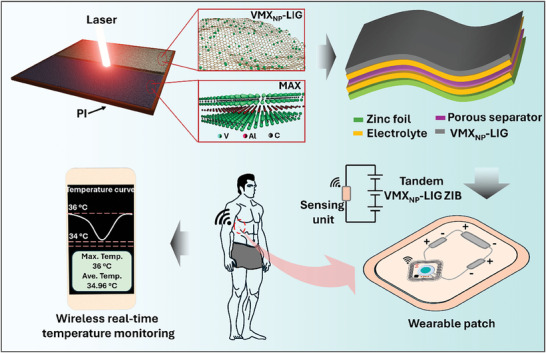
Schematic illustration of fabrication and application of flexible VMX_NP_‐LIG ZIB to track human body temperature through a wireless communication system.

Next, flexible Zn‐ion batteries (ZIBs) were constructed using Zn foil as an anode and our V‐rich MXene NPs (VMX_NP_) linked with laser‐induced graphene (VMX_NP_‐LIG) as a cathode. To demonstrate real‐time body temperature monitoring, the VMX_NP_‐LIG ZIB powered a commercial temperature sensing patch that monitors the real‐time human body temperature via wireless communication to a smartphone (see **Scheme** **1**). Before delving into the detailed evaluation of EC performance and healthcare applications, we first examine the morphological, microstructural, and chemical bonding environment of VMX_NP_‐LIG, detailed discussion in the following sections.

### 2.1 Synthesis and Characterization of VMX_NP_ Functionalized LIG Sheets

**Figure 1:**
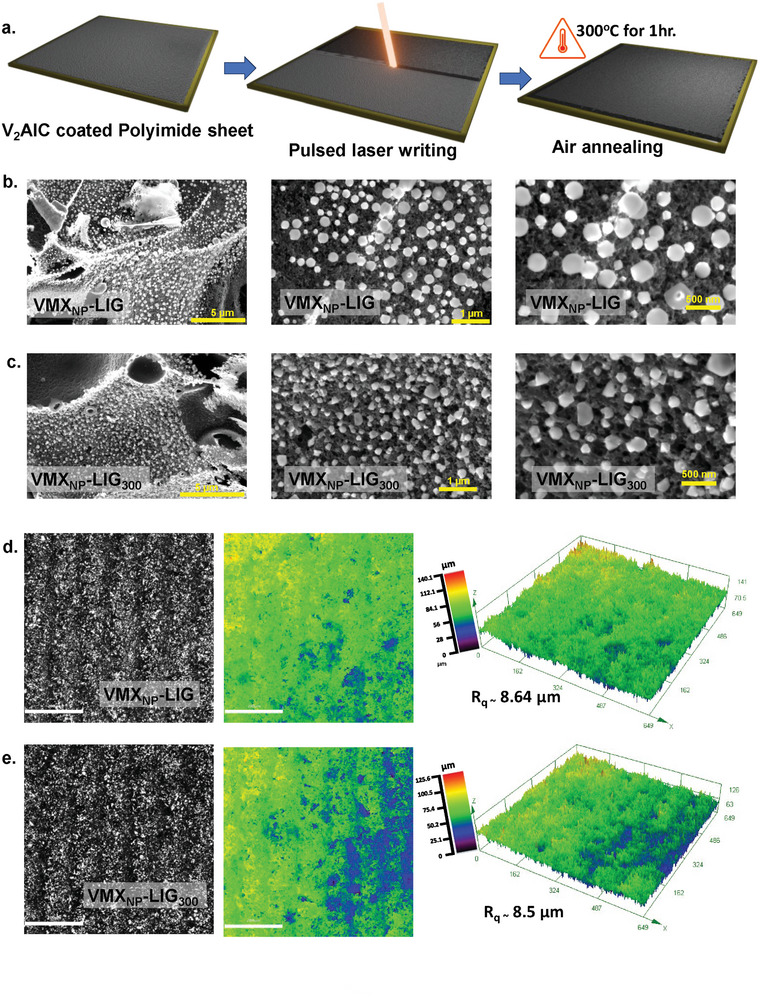
Scheme for the fabrication of VMX_NP_‐LIG_300_ cathode using pulsed laser and morphological characterization. a) Scheme of the microfabrication steps; single step pulsed laser writing on V_2_AlC coated PIS followed by air annealing at 300 °C. Different magnified SEM top view of b) VMX_NP_‐LIG and c) VMX_NP_‐LIG_300_ films where a uniform decoration of VMX_NP_ across the LIG surface can be seen. CLSM optical image along with the corresponding 2D and 3D color profiles of d) VMX_NP_‐LIG, e) VMX_NP_‐LIG_300._ Scale bar 200 µm.

**Figure** **1**a depicts the schematic diagram for preparing VMX_NP_‐decorated LIG. Initially, V_2_AlC was drop‐cast onto a flexible PIS, then subjected to pulsed laser writing (Nd:YAG) using a defocused lasing technique (Figure S1, Supporting Information). This method enables the adjustment of size of the laser's spot while preserving a constant dot density, allowing for multiple lasers passes in a single run and enhancing processing speed. The laser process facilitates the in situ decoration and bonding of V‐rich oxide NPs within the 3D porous LIG network. Upon exposure to an infrared laser pulses, V_2_AlC‐coated PIS quickly absorbs the infrared energy, resulting in a rapid increase of sheet temperature (around 2000 K) within a submillisecond timespan.^[^ ^34^ ^]^ This results in the creation of a 3D porous interconnected LIG structure due to carbonized steam production from the PIS. Simultaneously, the short time laser pulse energy can cause enough mechanical and thermal stress on V_2_AlC to break the V_2_AlC sheets into spherical V‐rich oxide NPs.^[^ ^35^ ^]^ Finally, the electrodes were air‐annealed at 300 °C (VMX_NP_‐LIG_300_) before being used as ZIBs.

The successful formation of V‐rich oxide NPs attachment over LIG was witnessed by scanning electron microscopy (SEM). Figure 1b illustrates the uniform distribution of V‐rich oxide NPs over the 3D interconnected network of LIG and the size of the NPs ranges from µm to sub‐nanometer level. The high‐magnification SEM images make the particle size more apparent. However, there is not much observable change in the morphology of the postheat‐treated VMX_NP_‐LIG_300_ sample. For comparison, SEM images of LIG, V_2_AlC powder, and laser‐induced V_2_AlC powder are shown in Figures S2 and S3 (Supporting Information), revealing the porous 3D interconnected structure of LIG, smooth and rough stacked layers of V_2_AlC and laser‐induced V_2_AlC respectively. To measure the dimensions of the NPs, tapping mode atomic force microscopy was performed, revealing nanoparticle diameters ranging from ≈30 to 200 nm (Figure S4, Supporting Information). This type of heterostructure, with uniformly distributed V‐rich oxide NPs over graphene, improves space utilization as well as effectively prevents the self‐restacking problem of graphene sheets.

Figure 1d,e displays optical images of VMX_NP_‐LIG and VMX_NP_‐LIG_300_, respectively, acquired with a confocal laser scanning microscope (CLSM). The topography is shown with 2D and 3D color mapping, where the discrete colors indicate the varying height profiles. Surface roughness (*R*_q_) of the VMX_NP_‐LIG and VMX_NP_‐LIG_300_ films was calculated as ≈8.64 and ≈8.5 µm, respectively. After undergoing postannealing treatment, the film surfaces exhibit increased smoothness, resembling the findings of a recent study on sp^3^ carbon surfaces.^[^ ^36^ ^]^ Such surface modifications are indicative of the influence of annealing on enhancing the quality and uniformity of the film surfaces, potentially offering improved performance and functionality in EC applications.

**Figure 2:**
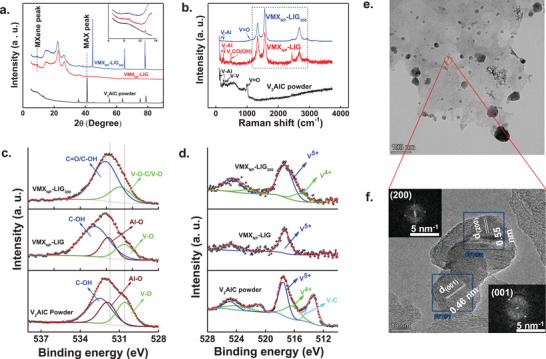
Physical characterization of VMX_NP_‐LIG and VMX_NP_‐LIG_300_ cathode. a) XRD spectra were obtained for V_2_AlC powder (black), VMX_NP_‐LIG (red), and VMX_NP_‐LIG_300_ (blue), with the inset displaying a magnified region within the 2*θ* range of 4°–16°. b) Raman spectra of V_2_AlC powder (black), VMX_NP_‐LIG (red), and VMX_NP_‐LIG_300_ (blue). c) O 1s and d) V 2p high‐resolution XPS spectra of V_2_AlC powder, VMX_NP_‐LIG, and VMX_NP_‐LIG_300_. e) TEM of VMX_NP_‐LIG_300_. Scale bar 100 nm. f) HRTEM image of VMX_NP_ revealing the presence of V_2_O_5_ NPs. Scale bar 10 nm.

We examined the phase transition of V_2_AlC following laser treatment using X‐ray diffraction (XRD) patterns (**Figure** **2**a). The peaks from V_2_AlC powder align well with previously reported V_2_AlC phases, indicating no impurities.^[^ ^31^ ^]^ For VMX_NP_‐LIG and VMX_NP_‐LIG_300_, we observe some remaining V_2_AlC alongside a new peak around 2*θ* ≈ 9.40°, attributed to the (0002) planes of V_2_C. This peak can be assigned to the (0002) planes of MXene and translated to c‐LP of 19.76 Å confirming the synthesis of V_2_C.^[^ ^37^ ^]^ Meanwhile, the residual peak around 2*θ* ≈ 41.2° indicates the V_2_AlC phase has not been completely converted to V_2_C MXene. It is noteworthy that Van der Waals or hydrogen bonds weakly bind the multilayered V_2_C MXene;^[^ ^38^ ^]^ laser interaction weakens these bonds, causing delamination and mechanical stress on the MXene sheets, leading to their fragmentation into spherical NPs.

Raman spectroscopy was used next to validate the XRD findings, investigate the degree of disorder in the graphitic region, and verify the different phases of VMX_NP_ (Figure 2b). The peak around ≈150 and ≈245 cm^−1^ of V_2_AlC powder corresponds to the in‐plane vibration of V and Al atoms. The other two peaks around ≈274 and ≈335 cm^−1^ correspond to V atoms' in‐plane and out‐of‐plane vibration, respectively. The peak at ≈1000 cm^−1^ is revealing the stretching mode vibration of V═O bonds. Note that for VMX_NP_‐LIG and VMX_NP_‐LIG_300_ in‐plane vibrational V‐Al peak is still there with an additional peak around ≈483 cm^−1^ which can be assigned to the out‐of‐plane vibration of the V atom model (V_2_CO(OH)) of V_2_C MXene. It seems that VMX_NP_‐LIG and VMX_NP_‐LIG_300_ contain both MAX (V_2_AlC) and MXene (V_2_C) phases respectively.

To better understand graphene defects due to the incorporation of V‐rich oxide NPs and postannealing treatment, we conducted Raman spectra analysis of VMX_NP_‐LIG and VMX_NP_‐LIG_300_. The spectra were fitted with a Lorentzian function in the region of 1000–3100 cm^−1^ (Figure S5, Supporting Information). Fitting the 2D band with a single Lorentzian peak revealed the existence of a few graphene layers oriented along the *c*‐axis, yielding FWHM values of 72 and 70 cm^−1^ for VMX_NP_‐LIG and VMX_NP_‐LIG_300_, respectively. Notably, there was a significant decrease in the I_D_/I_G_ value for VMX_NP_‐LIG_300_ (I_D_/I_G_ ≈ 0.51) compared to pristine VMX_NP_‐LIG (I_D_/I_G_ ≈ 0.79), indicating a lower defect concentration in the carbon lattice of VMX_NP_‐LIG_300_ relative to pristine VMX_NP_‐LIG. The detailed fitting results are included as Table S1 (Supporting Information).

XRD and Raman analyses reveal significant changes in the surface composition of VMX_NP_‐LIG_300_ after laser writing and subsequent annealing treatment. To validate these surface chemical alterations, X‐ray photoelectron spectroscopy (XPS) measurements were carried out. The survey spectra (Figure S6, Supporting Information) reveal the presence of V, C, O, and Al in all three samples. Notably, the XPS Al 2p peak exhibits a progressive decrease from V_2_AlC to VMX_NP_‐LIG_300_. Moreover, a substantial increase in the V/Al ratio, from 0.25 for V_2_AlC to 1.20 for VMX_NP_‐LIG_300_, is observed. This reduction in Al atoms further confirms the gradual conversion from V_2_AlC (MAX) to V_2_C (MXene) phase during laser treatment aligning well with the findings from XRD analysis. The detailed atomic percentage (At%) extracted from the XPS survey scan is presented in Table S2 (Supporting Information).

The high‐resolution C 1s spectra (Figure S7, Supporting Information) reveal the four peaks of V_2_AlC with binding energies of V–C/Al–C (≈282.7 eV), C–C (≈284.75 eV), C–OH (≈286.4 eV) and O–C═OH (≈289 eV).^[^ ^39^ ^]^ Post laser process, the peak identified to V–C/Al–C disappeared for VMX_NP_‐LIG (Figure S8, Supporting Information) and VMX_NP_‐LIG_300_ (Figure S7, Supporting Information). This implies the possibility of the formation of VO*_x_*‐C composite in case of VMX_NP_‐LIG and VMX_NP_‐LIG_300_ which is verified further by analyzing the O 1s core level spectra (Figure 2c). O 1s spectra demonstrated three peaks: V–O (≈530.6 eV), Al–O (≈531.8 eV), and C–OH (≈532.6  ± 0.2 eV) for V_2_AlC and VMX_NP_‐LIG sample.^[^ ^40^ ^]^ Note that the Al–O peak is absent in VMX_NP_‐LIG_300_ while the other two peaks shifted to the higher binding energy value. In case of VMX_NP_‐LIG_300_ two separate components can be clearly identified in the O1s peak: V–O–C/V–O (≈531 eV) and C═O (≈532.1 eV).^[^ ^41^ ^]^ Similar types of covalent bonding were observed before with vanadium, manganese, and titanium‐based transition metal oxides.^[^ ^34^, ^41^, ^42^ ^]^ The strong link that the V–O–C bond creates between VO*_x_* and LIG improves the charge transfer mechanism at their interface.

The laser energy‐induced thermal oxidation of V_2_AlC surface leads to the valence state change of V atoms as well which is studied in detail by analyzing the V 2p core level spectra (Figure 2d). V 2p spectrum of pristine V_2_AlC is deconvoluted into three distinct peaks; V–C (≈513.4 / ≈521.1 eV for V 2p_3/2_/V 2p_1/2_), V^4+^ (≈516.3/≈523.7 eV for V 2p_3/2_/V 2p_1/2_) and V^5+^ (≈517.5/≈524.8 eV for V 2p_3/2_/V 2p_1/2_) which is in agreement with previous findings.^[^ ^32^ ^]^ Post laser treatment the peak corresponding to V–C and V^4+^ state disappear and only V^5+^ (≈517.3/≈524.75 eV for V 2p_3/2_/V 2p_1/2_) dominates the V 2p spectrum. Single valence V is one of the bottlenecks to realizing large capacity.^[^ ^43^ ^]^ However, upon annealing the V^4+^ again arises along with V^5+^. These high valence V species can be identified as multivalent VO*_x_* arising from the postannealing method. Nonetheless, the at% ratio of V^5+^ to V^4+^ ($\frac{V^{5+}}{V^{4+}}$ ≈ 2.0), suggesting the V_2_O_5_ phase dominates in the VMX_NP_ particles (as seen in the SEM images) which is linked to the carbon network of LIG via V–O–C covalent bonding. The strong coupling between VO*_x_* and conductive carbon network has been proven as an effective way to greatly improve the performance of functional materials.^[^ ^44^ ^]^

The morphology and crystal structure of the VMX_NP_‐LIG_300_ hybrid were further characterized using transmission electron microscopy (TEM) and high‐resolution TEM (HRTEM). As depicted in Figure 2e, the VMX_NP_ are well distributed and decorated across the LIG network, exhibiting particle sizes ranging from ≈10 to 100 nm. HRTEM analysis provided insights into the layer spacing of VMX_NP_. The HRTEM profile of VMX_NP_ reveals distinct planar arrangements in different regions, as indicated by blue rectangles (Figure 2f). Notably, the lattice fringes in one region exhibit an interlayer spacing of 0.46 nm, corresponding to the (001) plane of V_2_O_5_, which represents a primary crystal plane growing outward along the *c*‐axis. In another region, lattice fringes with a spacing of 0.55 nm are observed, corresponding to the (200) plane of orthorhombic V_2_O_5_. The insets display this region's fast Fourier transform (FFT) diffraction pattern.

### 2.2 Electrochemical Performance of VMX_NP_‐LIG and VMX_NP_‐LIG_300_ Cathode‐Based AZIBs

**Figure 3:**
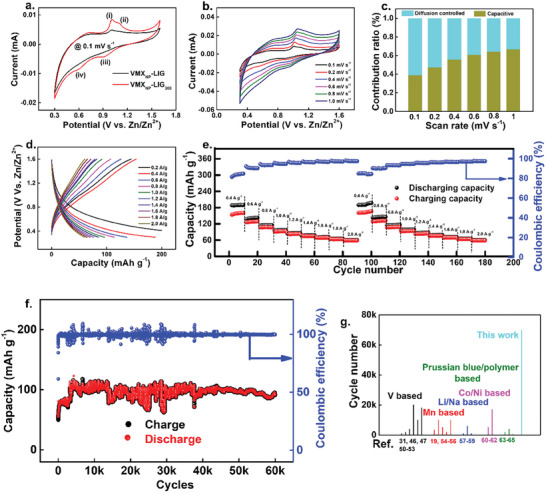
Electrochemical performance of VMX_NP_‐LIG and VMX_NP_‐LIG_300_ cathode‐based ZIBs in 2 m ZnSO_4_ electrolyte. a) CV curves of VMX_NP_‐LIG (black) and VMX_NP_‐LIG_300_ (red) within the potential window of 0.3–1.6 V. b) CV curves of VMX_NP_‐LIG_300_ with varying scan rates. c) Contribution proportions of the diffusion and capacitive component in the VMX_NP_‐LIG_300_ cathode. d) Typical charge‐discharge profile of VMX_NP_‐LIG_300_ cathode with varying current densities. e) Rate performance of VMX_NP_‐LIG_300_ cathode at varying current densities. f) Long‐term cyclic performance of VMX_NP_‐LIG_300_ cathode at 2 A g^−1^. g) The cycle life of aqueous ZIBs based on vanadium, manganese, lithium/sodium, cobalt/nickel, and polymers as reported in recent years.

To assess the EC performance of the VMX_NP_‐LIG_300_ cathode for ZIBs, we initially constructed our as‐prepared VMX_NP_‐LIG_300_ as cathode, paired with zinc foil as anode, utilizing a 2 m ZnSO_4_ aqueous electrolyte. As depicted in **Figure** **3**a, cyclic voltammetry (CV) results of VMX_NP_‐LIG and VMX_NP_‐LIG_300_ cathodes were attained at a scan rate of 0.1 mV s^−1^. Notably, VMX_NP_‐LIG_300_ exhibited a larger loop area compared to VMX_NP_‐LIG, indicative of its superior specific capacity. Furthermore, the post‐annealing treatment resulted in the emergence of two redox peak pairs between 0.4 and 1.2 V in (denoted as i, ii, iii, and iv) in the VMX_NP_‐LIG_300_ samples. With increasing scan rate, these peaks gradually broadened, with the oxidation peaks (peaks i and ii) shifting to higher potential and the reduction peaks (peaks iii and iv) shifting to lower potential. This phenomenon suggests enhanced pseudocapacitive behavior attributed to the stripping of V_2_C MXene and the appearance of multiple oxidations of V atoms, as corroborated by XPS analysis.^[^ ^41^, ^45^ ^]^

The surface capacitive effect of the VMX_NP_‐LIG_300_ cathode was assessed using the equation *i* = *aν^b^*,^[^ ^31^ ^]^ where *ν* stands for scan rate, *i* stands for current, and *a* and *b* are configurable parameters. The slope of the log(*i*) against log(*ν*) plot was used to calculate the value of *b* (refer to Figure S9 in the Supporting Information), where the diffusion‐controlled processes are indicated by *b* = 0.5, and capacitive‐controlled processes are indicated by *b* = 1.0.

The calculated *b* values for peaks i–iv were 0.56, 0.68, 0.62, and 0.57, respectively, suggesting a synergistic charge storage mechanism in which both diffusion and capacitive behaviors are present within the VMX_NP_‐LIG_300_ heterostructures, with diffusion‐controlled behavior being the dominant process.

We employed the following formulae to determine the additional contribution of the total capacity at a given scan rate (from peak iii) to evaluate the capacity ratio from capacitive (*k*_1_ *v*) and diffusion‐ (*k*_2_ *v* ^1/2^) controlled process at a particular voltage during cycling^[^ ^46^ ^]^

(1) $$i=k_{1}\nu +k_{2}v^{1/2}\;\mathrm{and}\;i/v^{1/2}=k_{1}v^{1/2}+k_{2}$$

Note that as the sweep rate increases, the capacitive contribution to the storage mechanism progressively rises, while the diffusion‐controlled process dominates the overall contribution at lower scan rates. For instance, at 1 mV s^−1^, the capacitive component accounts for ≈66% of the overall contribution, significantly enhancing the electrode's capacitive ratio at higher scan rates. As a result, the short ion diffusion path and high capacitance brought about by quick electron transfer are responsible for VMX_NP‐_LIG_300_’s better performance. Hence, the outstanding high‐rate performance observed in VMX_NP_‐LIG_300_ systems can be attributed to its notable capacitive‐controlled kinetics process. This feature sets it apart from previously documented vanadium‐based cathode materials characterized by crystalline phases for ZIBs.^[^ ^47^, ^48^, ^49^ ^]^

The comparative galvanostatic charge/discharge (GCD) profiles (Figure S10, Supporting Information) between LIG, VMX_NP_‐LIG, and VMX_NP_‐LIG_300_ reveal the superior capacity output of VMX_NP_‐LIG_300_ over the other two electrodes. Figure 3d illustrates the typical GCD profiles of the VMX_NP_‐LIG_300_ cathode with varying current densities. Notably, a discernible charge plateau at ≈1.05/1.1 V aligns with the dominant oxidation peak i observed in the CV curve, indicative of the electrode's redox process and the intercalation of Zn^2+^ ions within the V_2_O_5_/V_2_C cathode. Operating within a voltage window of 0.3–1.6 V, the electrode achieves an impressive capacity of 200 mAh g^−1^ and energy density of ∼169 Wh kg^−1^ at 0.2 A g^−1^ (Figure 3d). Furthermore, the aqueous VMX_NP_‐LIG_300_/Zn battery demonstrates remarkable rate capability, as evidenced in Figure 3e, with discharge capacities of ≈188, 137, and 113 mAh g^−1^ observed at current densities of 0.4, 0.6, and 0.8 A g^−1^, respectively. Notably, the discharge capacity is swiftly recovered to ≈189, 142, and 114 mAh g^−1^ upon reverting to current values of 0.4, 0.6, and 0.8 A g^−1^, respectively, demonstrating strong structural stability and significant acceptance to Zn^2+^ intercalation/deintercalation processes. Particularly remarkable is the ultralong cyclic stability exhibited by the VMX_NP_‐LIG_300_/Zn battery, as depicted in Figure 3f, retaining reversible capacity even after 60 000 GCD cycles at 2 A g^−1^ suggesting a superstable structure during long‐term cycle. Here the capacity increases with increasing cycling numbers and capacity retention of ≈150% with ≈100% coulombic efficiency achieved after 60 000 cycles. The capacity increase with cycling numbers is likely attributed to the gradual activation of the electrode, resulting in the enhanced accessibility of EC sites and subsequent higher specific capacity.^[^ ^50^ ^]^ Furthermore, a comparison with various vanadium (V),^[^ ^31^, ^46^, ^47^, ^50^, ^51^, ^52^, ^53^ ^]^ manganese (Mn),^[^ ^19^, ^54^, ^55^, ^56^ ^]^ lithium (Li),^[^ ^57^ ^]^ sodium (Na),^[^ ^58^, ^59^ ^]^ nickel (Ni),^[^ ^60^, ^61^ ^]^ cobalt (Co),^[^ ^60^, ^61^, ^62^ ^]^ and polymer‐based aqueous ZIBs^[^ ^63^, ^64^, ^65^ ^]^ reported in recent literature (Figure 3g) underlines the supercompetitive EC performance of our ZIB, particularly in cyclic stability at high current density, surpassing all current state‐of‐the‐art aqueous ZIBs reported to date.

### 2.3 Charge Storage Mechanism

**Figure 4:**
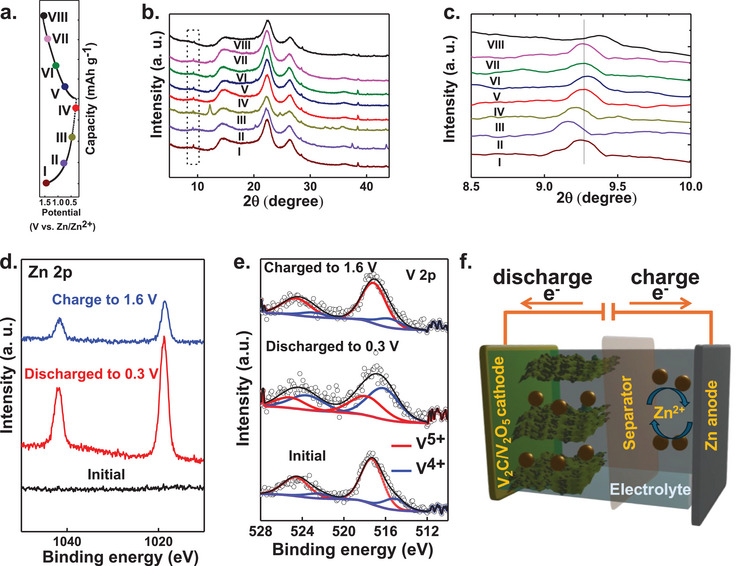
The charge storage mechanism of VMX_NP_‐LIG_300_/Zn battery. a) Charge–discharge curve of the VMX_NP_‐LIG_300_ cathode at 2 A g^−1^. b) Ex situ XRD spectra of the VMX_NP_‐LIG_300_ cathode at different state during charge–discharge process. c) Zoomed view of XRD spectra within the region of 2*θ* ≈ 8°–10°. Ex situ high‐resolution XPS spectra of the pristine VMX_NP_‐LIG_300_ cathode, along with the same cathode in its fully discharged and charged states, focusing on the d) Zn 2p region and e) V 2p region. f) An illustration of reversible Zn^2+^ intercalation/deintercalation mechanism.

To unveil the Zn^2+^ storage mechanism of the VMX_NP_‐LIG_300_/Zn battery, we conducted ex‐situ X‐ray diffraction (XRD) and X‐ray photoelectron spectroscopy (XPS) analyses on the VMX_NP_‐LIG_300_ cathode at various stages of the GCD process (**Figure** **4**a). The XRD patterns indicated the transformation of V_2_C/V_2_O_5_ to ZnV_2_O_6_ (PDF #1 534 159) during the initial discharging process (point II in Figure 4b), evidenced by the appearance of XRD peaks at 2*θ* ≈28.42°, ≈38.46°, ≈40.16 °, and ≈43.4°. Subsequent discharging to lower voltage led to the gradual prominence of these peaks, accompanied by the emergence of new phase peaks attributed to the layered Zn_3_(OH)_2_(V_2_O_7_): 2(H_2_O) (ZVOH; PDF # 2 006 776) with new peaks observed at 2*θ* ≈12.18°, ≈24.6°, ≈32.64°, ≈34°, and ≈35.66° (point III in Figure 4b). The structural evolution during subsequent charging followed a reverse trend to the discharge, with peaks corresponding to the ZVOH phases disappearing upon voltage reversal, indicating highly reversible intercalation/deintercalation of Zn^2+^ through ZVOH. The high reversibility was further corroborated by analyzing the shifting of the V_2_C (002) XRD peak (Figure 4c). During discharging from point I to point III, the 2*θ* value shifted from ≈9.26° to ≈9.2°, corresponding to an expansion of (002) interplanar distance (*d*_002_) from 9.54 to 9.59 Å; this value changed from 9.55 to 9.43 Å during the subsequent charging process (points V → VIII).

Additionally, ex situ XPS analyses of the cathode materials in their pristine state and fully charged/discharged states were performed to further explore the charge storage mechanism. As shown in Figure 4d, no Zn signal was detected at the pristine state. However, when charge to 1.6 V, two small Zn 2p core level peaks appeared, indicating successful pre‐ intercalation of a small amount of Zn^2^⁺ into the V_2_C/V_2_O_5_ interlayers following the phase evolution. When fully discharged, the Zn 2p peaks at ≈1021/≈1044 eV (2p_3/2_/2p_1/2_) showed a significant increase, indicating the intercalation of Zn^2+^ ions. The high‐resolution XPS spectra of V 2p is shown in Figure 4e. For the pristine VMX_NP_‐LIG cathode, V 2p spectra deconvoluted into a V^5+^ (2p_3/2_: 517.3 eV) along with a feeble V^4+^ component (2p_1/2_: 515.2 eV). At fully discharged condition, the V^4+^ component (2p_3/2_: 516.2 eV) became dominant, with a simultaneous appearance of a V^5+^ component (2p₃/₂: 518.1 eV) due to Zn^2^⁺ insertion. Upon charging, the V 2p spectrum returned to its original state, indicating a reversible EC redox process in the V_2_O_5_ framework driven by ongoing Zn^2^⁺ intercalation and deintercalation. The reversible Zn^2+^ intercalation/deintercalation mechanism is further illustrated in Figure 4f. During discharging Zn^2+^ in the electrolyte migrates to the cathode surface and intercalates in the V_2_C/V_2_O_5_ and during charging opposite reaction occurs.

### 2.4 Mechanism of Long Cyclic Stability

**Figure 5:**
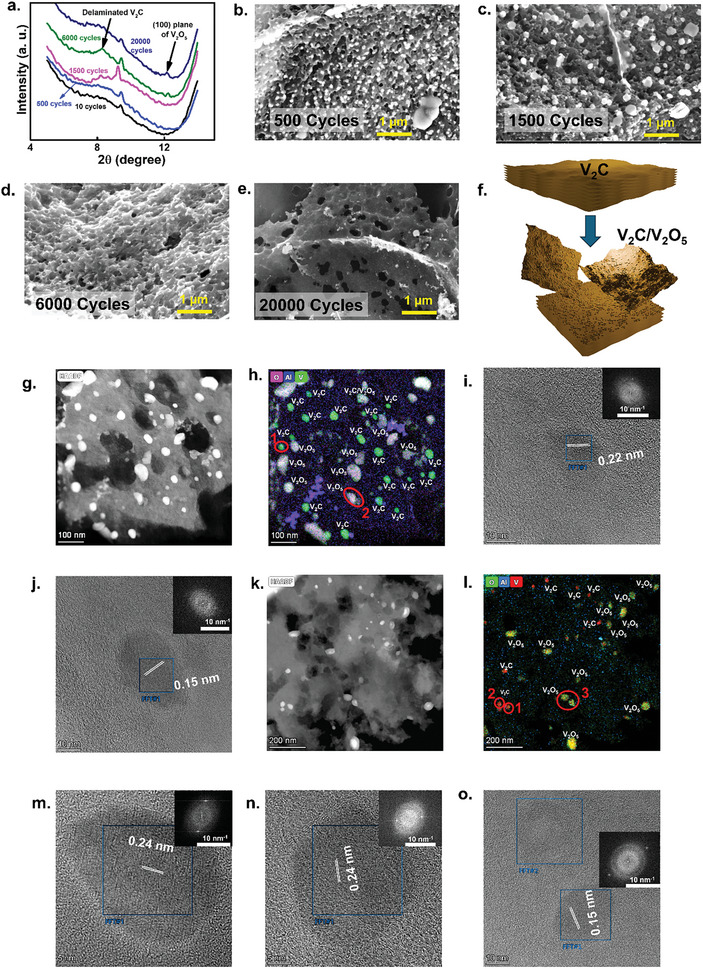
Mechanism of ultrastable cyclic performance of VMX_NP_‐LIG_300_/Zn battery. a) Ex situ XRD spectra of the VMX_NP_‐LIG_300_ cathode at different cyclic periods. b–e) SEM images of the V_2_C/V_2_O_5_ cathode after *n*th cycles. f) An illustration showing the probable mechanism of the phase and morphological evolution of the V_2_C cathode. HAADF images for g) VMX_NP_‐LIG_300_ cathode after the 6 k cycle (scale bar 100 nm). h) corresponding elemental mapping of V, O, and Al (scale bar 100 nm). i,j) HRTEM images of regions 1 and 2 of Figure 5h (scale bar 10 nm). Insets show their corresponding FFT spots. HAADF images for k) VMX_NP_‐LIG_300_ cathode after the 20 k cycle (scale bar 200 nm). l) Corresponding elemental mapping of V, O, and Al (scale bar 200 nm). m–o) HRTEM images of the regions 1, 2 (scale bar 5 nm), and 3 (scale bar 10 nm) of (l). Insets show their corresponding FFT spots.

To unveil the extraordinary capacity enhancement mechanism of the VMX_NP_‐LIG_300_ cathode, we conducted ex‐situ XRD and SEM analyses. The exceptional longevity of the battery under high current density is attributed to a multifaceted interplay of factors, among which three crucial observations stand out. First, we observed changes in the (002) interlayer distance (2*θ* ≈ 9.3°) of the cathode during cycling. The interlayer distance of VMX_NP_‐LIG_300_ remain same from 9.32 Å after 10 cycles to 9.5 Å (within experimental uncertainty) after 20 000 cycles, indicating synchronous Zn^2+^ intercalation upon discharge. Second, a broad peak emerged around 2*θ* ≈ 8° in the XRD patterns, credited to delaminated V_2_C fragments resulting from particle peeling during cycling (**Figure** **5**a).^[^ ^31^, ^38^, ^66^ ^]^ The stripping of layered materials substantially increases active sites in the cathode, contributing to enhanced capacity. Furthermore, this structural transition persisted throughout the cycling process, partially elucidating the capacity improvement observed in the V_2_C cathode through extended cycles. However, given the specificity and duration of the observed capacity improvement, cathode stripping alone is unlikely to be the dominant mechanism. Lastly, XRD analysis post 6000 GCD cycles revealed a new peak around 2*θ* ≈ 12.2°, attributed to the V_2_O_5_ phase's (100) plane.^[^ ^31^ ^]^

The intensity of the 12.2° peak increased progressively with cycling, while the neighbouring peaks at 8° and 9.3° (002 lattice of V_2_C) weakened, signaling a phase transition from V_2_C to V_2_O_5_ over the cycling duration.^[^ ^49^, ^67^ ^]^

The extended cycling mechanism was further confirmed through the morphological evolution of the cathode at different stages of cycling (Figure 5b–e). Initially, after 10 cycles, the morphology of VMX_NP_‐LIG_300_ closely resembled that of the pristine VMX_NP_‐LIG_300_ cathode (as depicted in Figure S11, Supporting Information). Distinct VMX_NP_ were clear throughout the three‐dimensional network of LIG. Up to 1500 cycles, minimal changes in morphology were observed (Figure 5b,c). However, after 6000 GCD cycles, a reduction in particle size was observed, attributed to the delamination of stacked few‐layered V_2_C. Subsequently, significant morphological changes were noted, with the V_2_C morphology becoming noticeably smoother, accompanied by the presence of distinct and densely packed granular particles as the cycle number exceeded over 20 000 (Figure 5f). This observation agrees well with XRD analyses and highlights the synergistic contribution of both pure cathode materials and the phase transition products to the overall capacity enhancement, leading to exceptional capacity retention over >60 000 GCD cycles.

The proposed mechanism for capacity enhancement, investigated by the preceding analyses, is depicted in Figure 5f. Initially, during the onset of the cycle, the insertion of Zn^2+^ ions results in the expansion of the layer spacing of V_2_C, facilitating subsequent ion insertion/extraction processes. As the cycling progresses, multi‐layered V_2_C sheets gradually delaminate from the original bulk material, providing additional active sites for ion accommodation, thus enhancing energy storage capacity, similar to observations in prior studies on other 2D materials.^[^ ^31^, ^46^ ^]^ Additionally, an increase in the valence state of V elements occurs as more V layers react with H_2_O in the electrolyte. This leads to the continuous generation of vanadium oxide, which contributes significantly to the overall capacity. Notably, V_2_O_5_, a prototypical battery‐type cathode material, exhibits remarkable EC activity and high capacity. The gradual formation of V_2_O_5_ and its replacement of V_2_C, which possesses only pseudocapacitive properties, further augments the overall capacity.

TEM images were captured after 6000 and 20 000 cycles to further examine the microstructural changes after extensive cycling. For the 6000‐cycle sample, the HAADF image (Figure 5g) reveals the presence of VMX_NP_ (bright spots) with random shapes. Corresponding energy dispersion spectrum (EDS) mapping shows the distribution of V, C, O, and Al elements (Figure 5h), with individual element mappings displayed in Figure S12 in the Supporting Information. STEM EDX mapping clearly distinguishes between the V_2_C and V_2_O_5_ NPs, consistent with XRD analysis. HRTEM images of regions 1 and 2 further illustrate V_2_C and V_2_O_5_ surrounded by LIG lattice planes. The crystal structure of V_2_C (Figure 5i) shows a specific arrangement with lattice spacing corresponding to the (103) plane at 0.22 nm, while the spacing of 0.15 nm corresponds to the (412) facets of the V_2_O_5_ phase (Figure 5i).

Similar HAADF images of the cathode after 20 000 cycles (Figure 5k) reveal VMX_NP_. EDS mapping shows the distributions of V, C, and O, but Al is absent (Figure 5l). Notably, the separate coexistence of V_2_C and V_2_O_5_ NPs is clearly visible, with a higher density of V_2_O_5_ NPs compared to V_2_C. This observation is consistent with XRD results, indicating the phase transition of V_2_C to V_2_O_5_ during repeated Zn intercalation/deintercalation processes. Individual elemental mappings are displayed in Figure S13 in the Supporting Information. HRTEM images of regions 4, 5, and 6 reveal that the interlayer spacing of the V_2_C (103) plane has slightly increased to 0.24 nm (Figure 5m,n), while for the V_2_O_5_ phase, the same (412) facets show an interlayer spacing of 0.15 nm (Figure 5o).

The exceptional long cycling life of the battery results from a complex interplay of factors, with two critical elements playing significant roles. First, the interlayer distance of the VMX_NP_ remains stable during GCD processes, unlike most other cathodes. Second, both the pristine cathode material and the phase transition product (V_2_O_5_ phase) exhibit excellent EC activity and high capacity, characteristic of a typical battery‐type cathode.

## 3 Theoretical Calculation

### 3.1 Stable ZIBs Enabled by the Interaction of Zn Atoms with V_2_C/V_2_O_5_ on LIG

**Figure 6:**
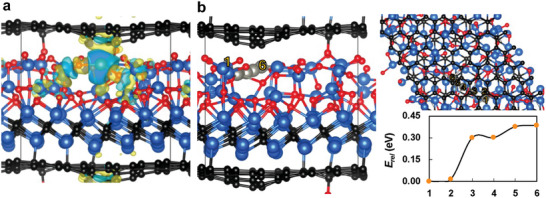
Interaction of Zn^2+^ on the surface of VMX_NP_‐LIG_300_ cathode. a) Charge density difference of Zn@graphene/V_2_C/V_2_O_5_. b) NEB path of Zn atom in graphene/V_2_C/V_2_O_5_. Diffusion barriers are calculated as relative energies with respect to the most stable adsorption site of Zn (number one). Carbon in black, vanadium in blue, oxygen in red, zinc in grey.

In order to reveal the interaction of Zn^2+^ on the surface of VMX_NP_‐LIG_300_ cathode, the adsorption between Zn^2+^ and graphene/V_2_C/V_2_O_5_ structures, density functional theory (DFT) calculations were performed to understand the structural and electronic properties of the MXene (V_2_C)/V_2_O_5_ decorated 3D graphene nanostructure. Since no structural models of the graphene/V_2_C/V_2_O_5_ were available in the crystallographic databases, our DFT models were built by layering the models of graphene, V_2_C, and V_2_O_5_ and optimizing the atomic positions and the whole cell (**Figure** **6**). In contrast to pristine graphene (Figure S14a, Supporting Information), which acquired a C–C bond distance of 1.42 Å, graphene layer of LIG/V_2_C/V_2_O_5_ (Figure S14c, Supporting Information) was curved with C–C bonds of 1.42–1.47 Å due to the graphene–V_2_C interlayer bonds of 2.08 Å and the graphene–V_2_O_5_ interlayer bonds starting from 1.44 Å. The bonds of V_2_C/V_2_O_5_ were rather disorganized with V–C bonds and V–O bonds of 1.92–2.10 and 1.62–2.22 Å, respectively. In comparison, the standalone V_2_C/V_2_O_5_ without interacting graphene (Figure S14b, Supporting Information) was more organized with V–C bonds and V–O bonds of 1.93–2.06 and 1.86–2.15 Å, respectively.

The interaction of the Zn atom with graphene/V_2_C/V_2_O_5_ (Figure 6a) was found to be relatively low with adsorption energies of Zn up to −1.23 eV. The nudged elastic band (NEB) of Zn in graphene/V_2_C/V_2_O_5_ showed energy barriers up to 0.40 eV (Figure 6b) indicating relatively easy diffusion of Zn within the system. In comparison, the adsorption energy of Zn with the standalone V_2_C/V_2_O_5_ (Figure S15a, Supporting Information) was calculated to be −2.15 eV, which suggests that the spacing between graphene and V_2_C/V_2_O_5_ is beneficial for the sufficient absorption and easy diffusion of Zn. The charge density difference of Zn@graphene/V_2_C/V_2_O_5_ showed the charge accumulation around the Zn atom and neighboring V and O atoms as reported also in,^[^ ^68^ ^]^ furthermore, the interaction of Zn with the π‐π‐cloud of graphene was demonstrated (Figure 6a; Figure S15, Supporting Information). The Bader analysis revealed the charge transfer from Zn to neighboring O atoms ranging from 0.69 to 1.13 e^−^ depending on the adsorption site.

In contrast to the semi‐metallic electronic structure of graphene (Figure S14, Supporting Information), the atom‐decomposed DOS of V_2_C/V_2_O_5_ showed the metallic electronic structure with the electron states at the Fermi level, implying high electronic conductivity in agreement with the amorphous V_2_O_5_@rGO^[^ ^69^ ^]^ (Figures S14 and S15, Supporting Information). Notably, more states appeared at the Fermi level of Zn@graphene/V_2_C/V_2_O_5_ as compared to standalone V_2_C/V_2_O_5_ due to the graphene layer, indicating even higher electronic conductivity (Figures S14 and S15, Supporting Information).

### 3.2 Flexible ZIB for Real‐Time Track of Human Body Temperature

**Figure 7:**
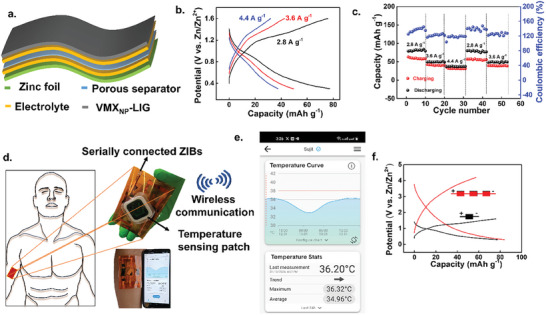
Electrochemical performance of VMX_NP_‐LIG_300_ cathode‐based ZIBs in PVA/ZnSO_4_ gel electrolyte. a) Schematic illustration of flexible ZIB. b) Typical charge‐discharge profile of VMX_NP_‐LIG_300_ cathode with varying current densities. c) Rate performance of VMX_NP_‐LIG_300_ cathode at varying current densities. d) Schematic illustration of real‐time tracking of human body temperature via wireless communication to a smartphone. Here temperature sensing patch is integrated into three serially connected ZIBs. e) Temperature profile of the two healthy male participants over a period of 24 hours. f) Charge–discharge profile of VMX_NP_‐LIG_300_ cathode of series‐connected flexible ZIBs.

The state of art flexible batteries demonstrate a variety of device configurations feature high conformability.^[^ ^7^ ^]^ By assembling the flexible VMX_NP_‐LIG_300_ cathode, and a flexible Zn anode with a PVA/ZnSO_4_ gel electrolyte sandwich between them a flexible ZIB was fabricated (**Figure** **7**a). GCD profiles of the flexible VMX_NP_‐LIG_300_/ZIB at varying current densities are depicted in Figure 7b. Notably, distinct charge plateaus are observed around ≈1.15 and ≈0.7 V, indicative of the redox mechanism of the electrode and the insertion of Zn^2+^ ions into the V_2_O_5_/V_2_C cathode. Operating within a potential window of 0.3–1.6 V, the electrode exhibits a noteworthy capacity of ≈77 mAh g^−1^ at a current density of 2.8 A g^−1^ (Figure 7b). Furthermore, the quasi‐solid‐state VMX_NP_‐LIG_300_/Zn battery demonstrates excellent rate capability, as illustrated in Figure 7c, where discharge capacity swiftly recovers upon reverting to different current densities, highlighting the electrode's remarkable structural stability and significant tolerance to Zn^2+^ intercalation/deintercalation processes.

While lithium‐ion batteries remain the preferred choice for biomedical applications, safety concerns persist, especially in scenarios where flexible batteries undergo frequent deformations in direct contact with the human body. Addressing these concerns, water‐based gel electrolytes coupled with both nontoxic anodes and cathodes offer enhanced safety and environmental friendliness, mitigating the drawbacks associated with conventional lithium‐ion batteries. In pursuit of real‐world applications, particularly in the biomedical domain, we integrated a precise temperature sensor patch with our quasi‐solid‐state flexible VMX_NP_‐LIG_300_/Zn battery (Figure 7c). This temperature sensor, powered by flexible serially linked VMX_NP_‐LIG_300_/Zn batteries, was affixed to the wrist of human subjects to monitor body temperature changes. The VMX_NP_‐LIG_300_/Zn batteries, along with the integrated temperature sensor patch, accurately detected variations in body temperature over time. The recorded body temperature (in °C) data were conveniently transferred to wireless NFC‐compatible handheld devices or smartphones for analysis. Over 24 h, body temperature readings were obtained from two healthy male participants (aged 36 and 30) using our VMX_NP_‐LIG_300_/Zn batteries and integrated temperature sensor patch, with the smartphone recording temperature data illustrated in Figure 7e. Employing three serially connected flexible batteries as the power source for the temperature sensing patch, we achieved a stable output voltage of 4.2 V at 2.8 A g^−1^ (Figure 7f), along with a steady open circuit potential (OCP) of 3.1 V (Figure S16, Supporting Information). To evaluate the mechanical durability of our flexible ZIBs, the device performance was tested under different bending conditions and the associated voltage profile is shown as Figure S17a (Supporting Information). The discharge capacity gradually reduced from ≈97 mAh g^−1^ to ≈87, ≈84, ≈81, ≈79 as the device was bent from a slight angle (45°) to an extreme angle (180°). The flexible ZIB maintains ≈80% of its initial capacity even under extreme bending conditions, demonstrating the excellent mechanical stability of the device. Moreover, the device endurance test was conducted over 2000 discharge/charge cycles in a fully (180°) bent configuration (Figure S17b, Supporting Information) where ≈50% capacity decay was seen during the initial 800 cycles, after which the device was stable till 2000 cycles. The excellent mechanical flexibility of VMX_NP_‐LIG_300_/Zn batteries positions it as a promising contender for use in wearable electronics.

Given that body temperature serves as a crucial vital sign, and temperature measurement is among the primary methods for diagnosing illness, the continuous temperature tracking demonstrated by our portable integrated device underscores its potential for discreetly capturing physiological signals and wirelessly monitoring personal health conditions in real‐time, offering enhanced stability and reliability. Many MXenes possess excellent structural properties, but their limited stability in open environments restricts their use in wearable devices. In this study, we demonstrated an MXene‐based heterostructure (e.g., V₂C/V₂O₅) with ultralong stability enabled by phase transitions in cathode materials, highlighting its potential for wearable electronics. Furthermore, the electrode design process utilizing laser techniques enhances adaptability to nonplanar surfaces, facilitating seamless integration into flexible and stretchable devices. Overall, this work addresses material stability and scalability challenges by leveraging selective materials and advanced design techniques.

## 4 Conclusions

In summary, this study presents the development of an exceptionally stable AZIB through cathode engineering. By introducing V_2_C/V_2_O_5_ NPs covalently bound to the 3D network of laser‐induced graphene (LIG) not only keeps structural integrity but inhibits V dissolution while also facilitating charge transfer. This results in outstanding durability and impressive capacity output, with a specific capacity of ≈200 mAh g^−1^ at 0.2 A g^−1^, which is remarkable for aqueous‐based ZIBs. Moreover, we observe an unusual capacity enhancement of ≈150% even after 60 000 GCD cycles. The stripping of V_2_C MXene during repetitive cycling, exposing more active sites for reactions, contributes to this enhancement, although the main mechanism is the phase evolution of the V_2_C to V_2_O_5_, which endlessly improves capacity by using an alternative chemical route, both the original cathode material and the products of this phase evolution subsidize to the capacity over long lifespan. This dual contribution is a notable advantage of our battery system. Importantly, our V_2_C/V_2_O_5_@LIG‐based ZIB shows promising applications in wearable bioelectronics for health monitoring systems. The extended cycle life is particularly significant when compared to lithium‐ion batteries, which face challenges related to lifetime and safety concerns. In applications such as biomedical grafts, microsensors or RFID tags where substitute or repair is impractical, our ZIB can be seamlessly incorporated into bioelectronic devices, enabling the creation of highly efficient, autonomous real‐time health monitoring devices. As a proof of concept, we have integrated our ZIB with a temperature‐sensing patch to wirelessly monitor and track the real‐time body temperature of a human subject. Developing an ultrastable V_2_C/V_2_O_5_@LIG‐based ZIB can be suitable for future remote healthcare (“telehealth”) applications, including breathing monitoring, heartbeat tracking, and motion detection, which demand a reliable long‐term power supply for biosignal monitoring and transmission. Additionally, demonstrated advanced electrode design techniques using laser processing, which enhances design precision and scalability‐key factors for the commercialization of wearable electronic devices. Overall, this work demonstrates the potential of low‐cost V_2_AlC for ZIBs and offers an effective strategy to achieve ultra‐long cycle life battery performance through appropriate phase transition in cathode materials.

## 5 Experimental Section

### Materials

V_2_AlC MAX powders were obtained from Laizhou Kai Kai Ceramic Materials Co. Ltd. (Hong Kong S.A.R.). PIS (0.005″) were sourced from Fiedler Scientific Instruments, Czech Republic. Zinc foil and poly(vinyl alcohol) were acquired from Sigma‐Aldrich (Merck, Germany). The temperature‐sensing patch was purchased from an online retailer in Austria. All materials were utilized as received, without any additional purification or modification.

### Fabrication of the ZIB Cathode

VMX_NP_‐LIG cathode was fabricated using a diode‐pumped solid‐state Nd:YAG laser (Oxford Lasers A‐Series) operating at a wavelength of 532 nm. Initially, V_2_AlC was spin‐coated onto a PIS at 300 rpm for 60 s. The VMX_NP_‐LIG hybrid electrode was then created through defocused laser writing on the V_2_AlC‐coated PIS. The laser parameters were as follows: power of 3 W, scanning speed of 50 mm^−1^ s, pulse frequency of 7 kHz, resolution of 0.5 µm, and a central wavelength of 532 nm. After laser processing, the VMX_NP_‐LIG electrode was further annealed in air at 300 °C for 1 h in a tubular furnace.

### Materials Characterization

SEM images were obtained using the FEI VERIOS 460L. Elemental mapping was performed with the Mira 3 XMU (Tescan) equipped with an Oxford Instruments X‐MAX EDS detector. Surface chemical compositions were analyzed via XPS using a Kratos Analytical Axis Supra instrument with a monochromatic Al Kα (1486.7 eV) source. XPS spectra were calibrated to the C 1s peak at 284.8 eV and analyzed using CasaXPS software. Raman spectra were recorded over the range of 200–3000 cm^−1^ with a Witec Alpha 300R Raman spectrometer using a 532 nm laser. The surface roughness and height profiles of the films were assessed using a confocal laser scanning microscope (CLSM) (Olympus Lext OLS4100), employing a 405 nm wavelength laser during the measurements.

### Electrochemical Measurements

The aqueous ZIBs were constructed in a Swagelok cell configuration, employing V_2_C/V_2_O_5_‐LIG as the cathode, a 2 m ZnSO_4_ aqueous solution as the electrolyte, glass fiber separator (Whatman GF/B), and zinc metal foil (0.1 mm thickness, 99.95% purity, Sigma Aldrich) as the anode. After assembly, the cells were sealed in ambient conditions and allowed to stand for 12 h (to stabilize the OCP) before undergoing EC testing.

A PIS (25×75 mm) was coated with 1 mL of a 1 mg mL^−1^ V_2_AlC powder solution. Our cathode has an approximate area of 0.8 cm^2^, resulting in roughly 0.05 mg of active material (VMX_NP_) on the cathode. It is important to note that the LIG is used as a current collector.

EC measurements were conducted within a voltage range of 0.3–1.6 V. CV and GCD tests were carried out using a potentiostat/galvanostat (PGSTAT204, AutoLab Metrohm, Netherlands) connected to a computer equipped with Nova 2.1 software.

For quasi‐solid state ZIB, 1st The ZnSO_4_/poly(vinyl alcohol) (PVA) gel electrolyte was prepared as follows: 30 mL of ZnSO_4_ (1 m) aqueous solution and 3 g PVA (PVA‐117, *M*_w_ ≈ 145 000) were combined, and the mixture was heated to 85 °C for 4 h while being constantly stirred. Next, the quasi‐solid state ZIBs were prepared by uniformly applying a layer of polymer gel electrolyte onto both the V_2_C/V_2_O_5_‐LIG (cathode) and flexible Zn foil substrate (anode), followed by sandwiching anode and cathode with a glass fiber separator (Whatman GF/B) between them.

### Computational Details

The periodic boundary condition (PBC) calculations were performed using density functional theory (DFT) with the Perdew, Burke, and Ernzerhof (PBE) exchange and correlation functional^[^ ^70^ ^]^ and projected augmented wave (PAW) potentials^[^ ^71^, ^72^ ^]^ as implemented in the Vienna Ab initio Simulation Package (VASP).^[^ ^73^ ^]^ Dispersion contributions were introduced by the Grimme D3 empirical dispersion.^[^ ^74^ ^]^ The wave‐functions were expanded in the plane‐wave basis set with a minimum cut‐off of 500 eV. Both the geometry and the lattice optimizations were performed with the 3×3×3 Γ‐centered Monkhorst‐Pack *k*‐point mesh.^[^ ^75^ ^]^ All the optimized structures were converged to forces of less than 10^−2^ eV Å^−1^, and an electronic energy convergency criterion of 10^−5^ eV for each self‐consistent cycle. The tetrahedron method with the Blöchl corrections was used for energy calculations and density of states (DOS) plots. The nudged elastic band (NEB) method was used to determine the diffusion energy barriers of Zn.^[^ ^76^ ^]^ Charge analysis was performed using the Bader approach.^[^ ^77^ ^]^ The strength of the interaction was evaluated by adsorption energies as *E*_ads_ =  (*E*_system + Zn_ −  *E*_system_ − *E*_Zn_), where *E*_system + Zn_, *E*_system_, and *E*_Zn_, denoted total energies of whole Zn@LIG/V_2_C/V_2_O_5_, LIG/V_2_C/V_2_O_5_, and Zn atom respectively.

## Conflict of Interest

The authors declare no conflict of interest.

## Author Contributions

S.D. perceived and designed the experiments. S.D. prepared the cathodes and carried out the materials characterizations and ZIB fabrication and testing. S.D. and J.V.V. carried out biomedical assembly and related experiments using temperature‐sensing patches. M.O. and R.L. carried out the DFT calculations and theoretical analysis. J.M. performed the TEM measurements. S.D. wrote the manuscript. M.P. supervised the research. All authors discussed the results and approved the final version of the manuscript.

## Supporting information



Supporting Information

## Data Availability

The data that support the findings of this study are available from the corresponding author upon reasonable request.
