# Supplementary material for: Phase Transition Driven Zn‐Ion Battery With Laser‐Processed V2C/V2O5 Electrodes for Wearable Temperature Monitoring
Source: Small. 2024 Dec 29;21(7):2409987. doi: 10.1002/smll.202409987 (PMC11840462; doi:10.1002/smll.202409987)
Supplement: Supplementary file 1 — Supporting Information [file SMLL-21-2409987-s001.docx]

**Supporting Information**

**Phase Transition Driven Zn-Ion Battery with Laser-Processed V_2_C/V_2_O_5_ Electrodes for Wearable Temperature Monitoring**

Sujit Deshmukh^1^, [Jayraj V. Vaghasiya](https://scholar.google.com/citations?hl=en&user=Pps7xrgAAAAJ)^1^, Jan Michalička,^2^ Rostislav Langer,^4^ Michal Otyepka^3,4^ & Martin Pumera*^1,5,6^

^1^ Future Energy and Innovation Laboratory, Central European Institute of Technology, Brno University of Technology, Purkyňova 123, 61200 Brno, Czech Republic.

^2^Central European Institute of Technology, Brno University of Technology, Purkyňova 123, Brno, 61200, Czech Republic

^3^Regional Centre of Advanced Technologies and Materials, Czech Advanced Technology and Research Institute (CATRIN), Palacký University in Olomouc, Šlechtitelů 27, 783 71 Olomouc, Czech Republic

^4^IT4Innovations, VSB-Technical University Ostrava, 17. listopadu 2172/15, 708 00 Ostrava-Poruba, Czech Republic

^5^Faculty of Electrical Engineering and Computer Science, VSB - Technical University of Ostrava, 17. listopadu 2172/15, 70800 Ostrava, Czech Republic

^6^Department of Medical Research, China Medical University Hospital, China Medical University, No. 91 Hsueh-Shih Road, Taichung, Taiwan.

✉email: [martin.pumera@ceitec.vutbr.cz](mailto:martin.pumera@ceitec.vutbr.cz)


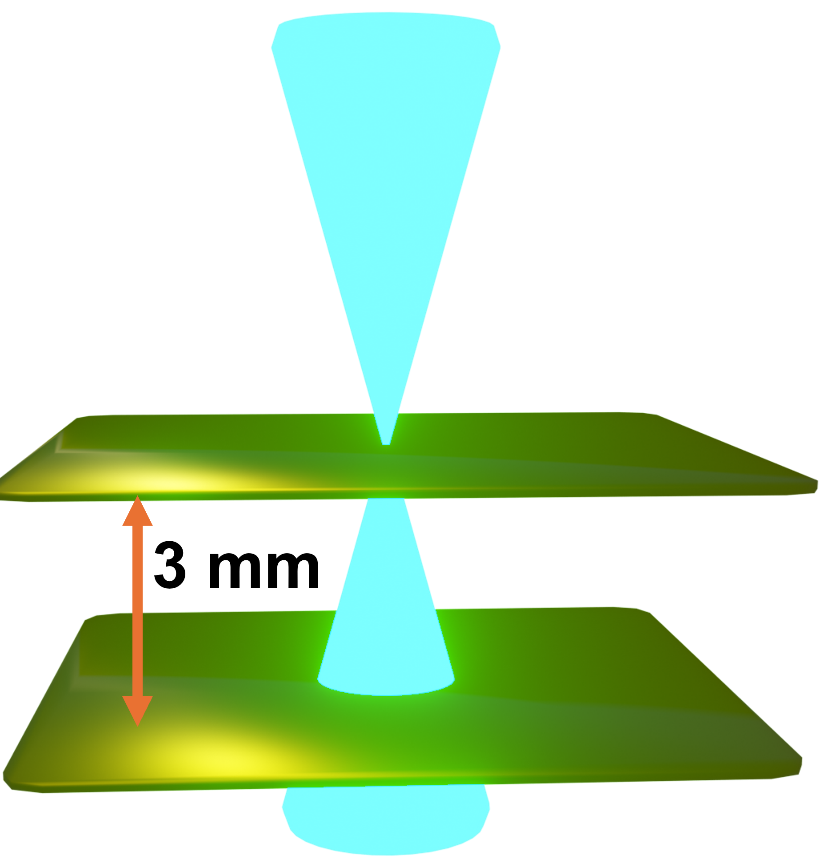


**Figure S1.** Defocused lasing method where the substrate was kept below 3 mm from the laser focal point.

The defocused method enables multiple lasing events during a single pass of the pulsed laser. This technique allows for easy adjustment of the laser spot size while preserving a consistent dot density. By lowering the substrate approximately 3 mm below the focal point, the spot size increases, facilitating multiple lasing events at a single location without altering the laser spot density. This defocused approach significantly improves processing efficiency by enabling multiple interactions in a single pass.


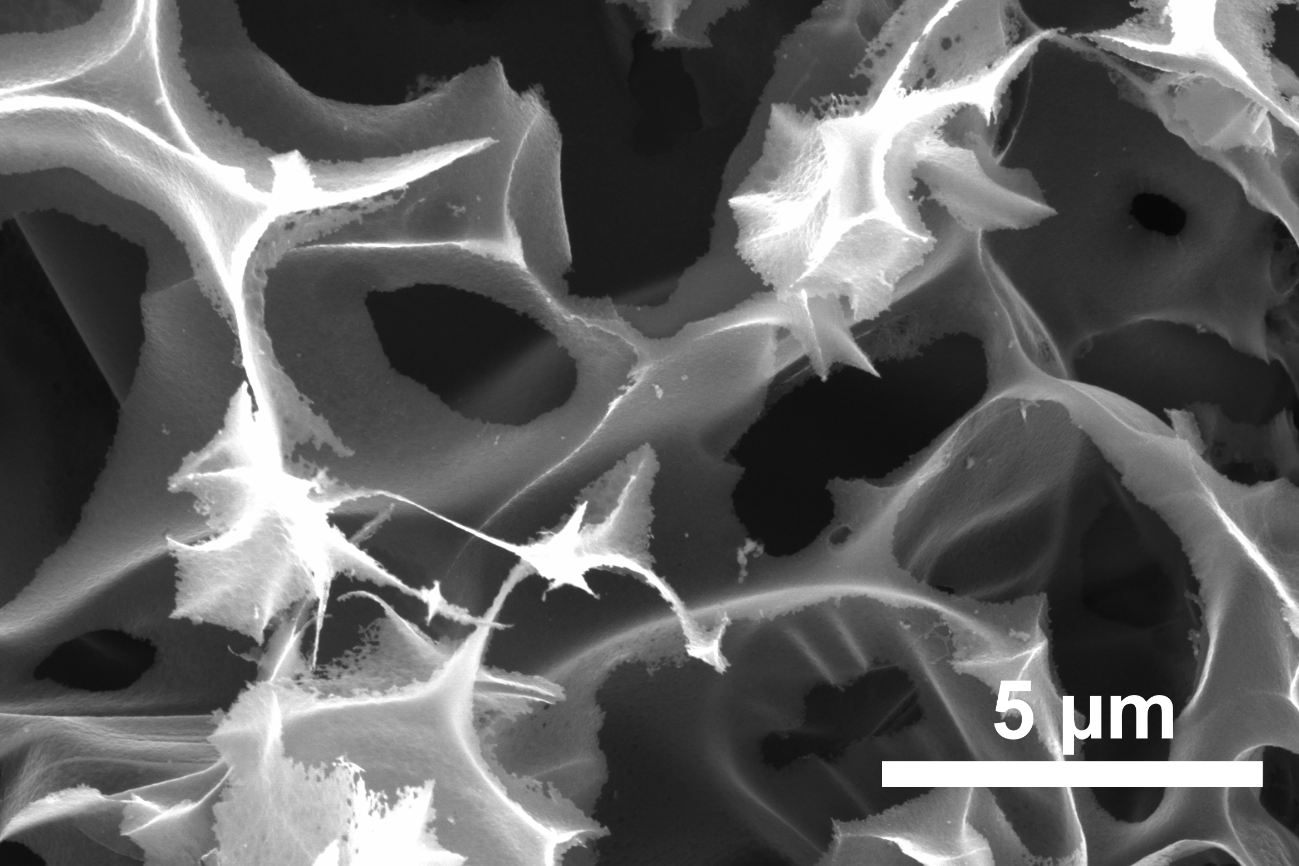


**Figure S2.** SEM top view of laser-induced graphene revealing 3D interconnected porous structures.


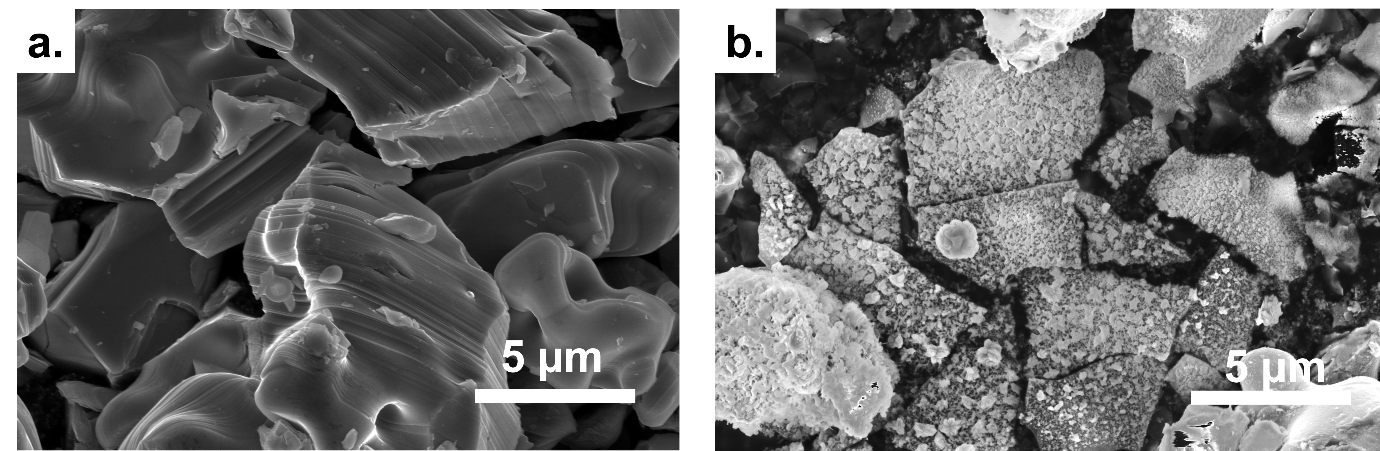


**Figure S3.** SEM top view of **(a)** smooth stacked layer of MAX (V_2_AlC) powder. **(b)** Rough laser-induced MAX (V_2_AlC) powder


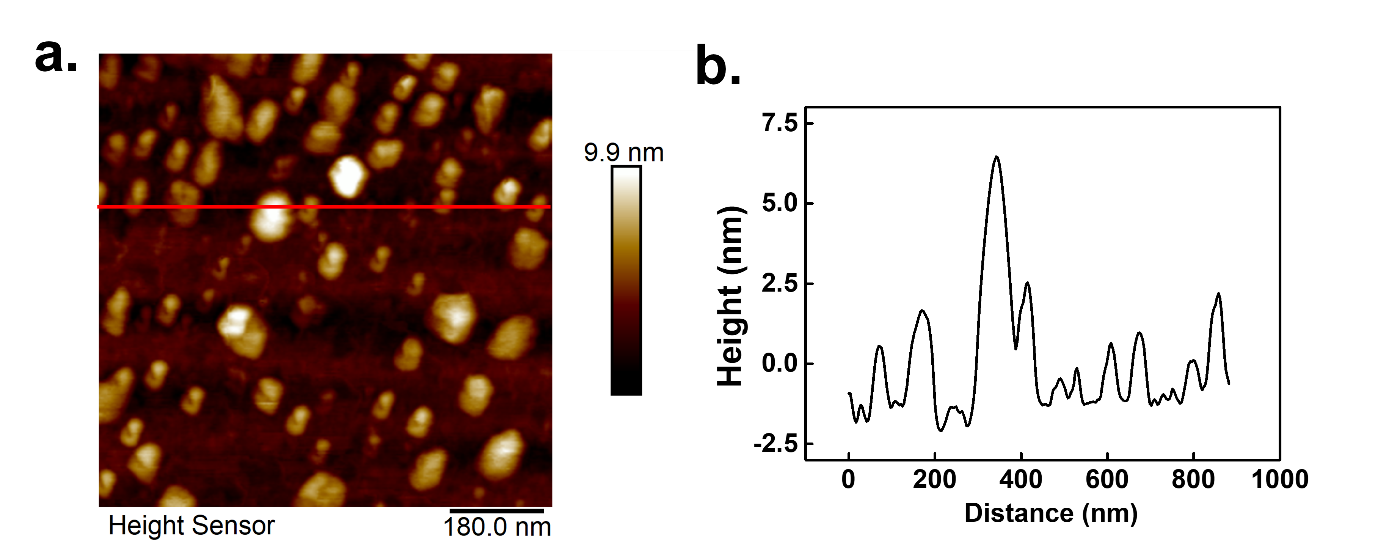


**Figure S4. (a)** AFM surface topography of VMX_NP_-LIG_300_ and corresponding **(b)** surface profile across the red line in (a). Random size distribution of particles is observed across the red line where particle size ranges from 30-200 nm.


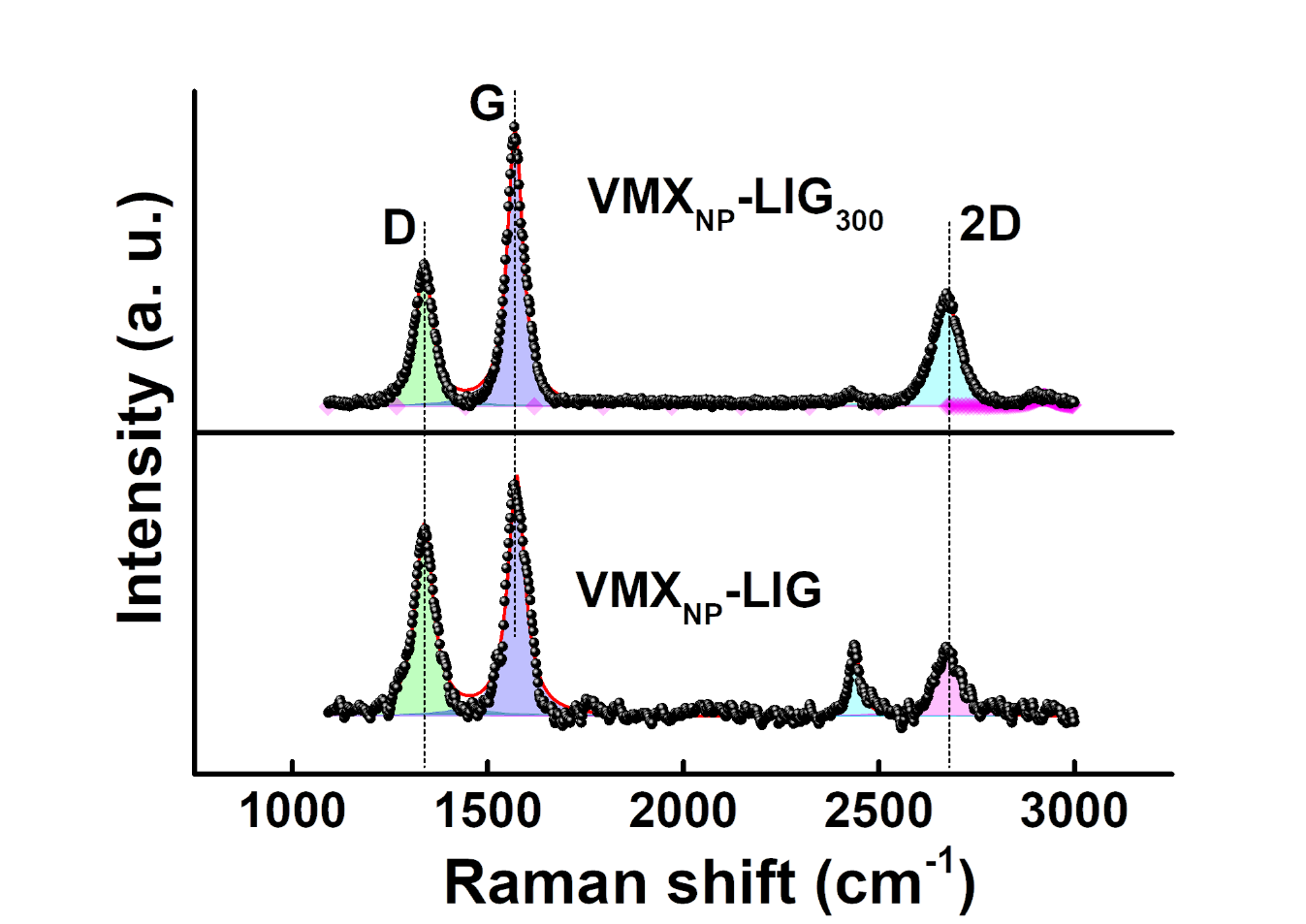


**Figure S5.** Fitted Raman spectra of VMX_NP_-LIG, and VMX_NP_-LIG_300_ within the range of 1000-3000 cm^-1^.


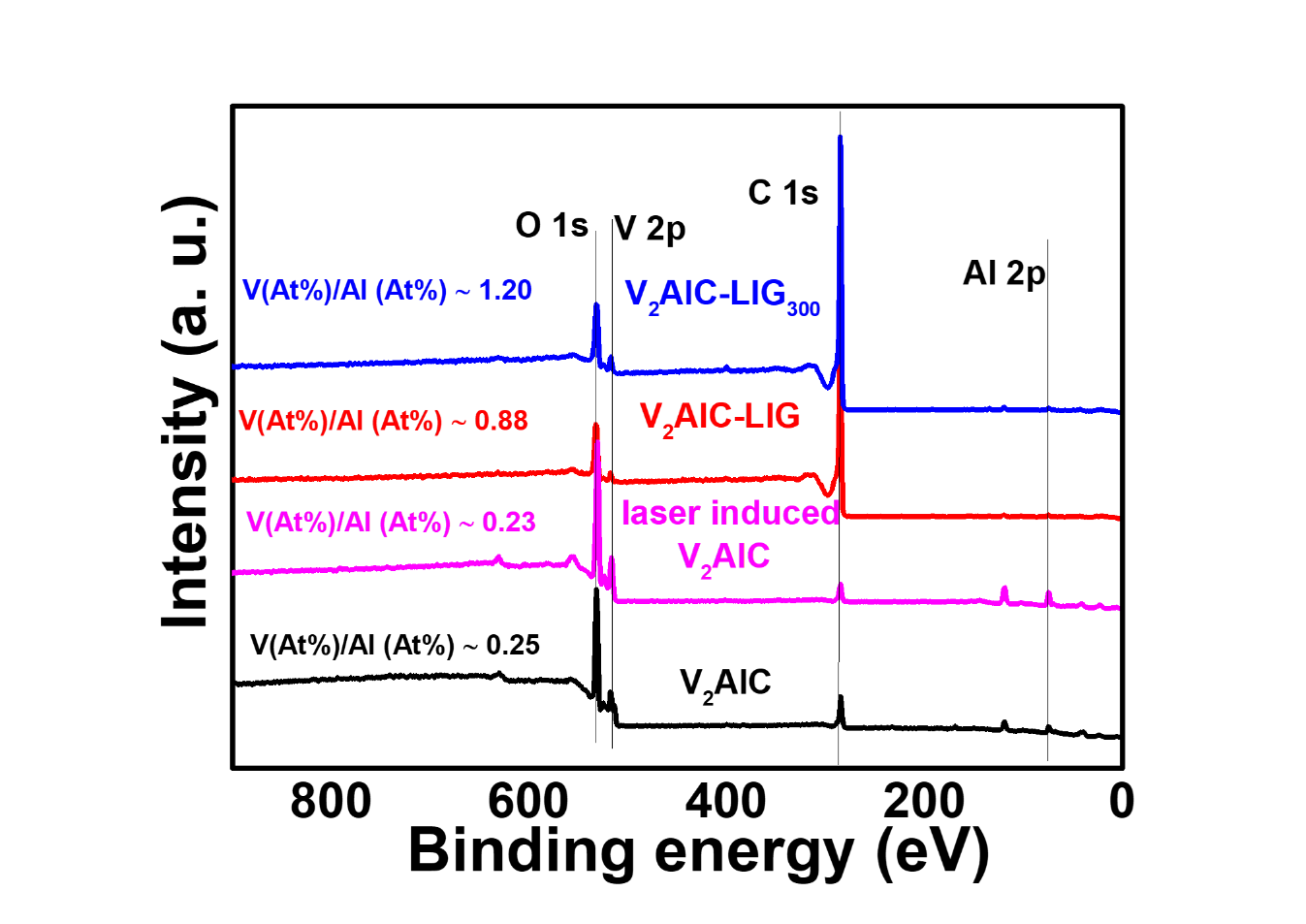


**Figure S6.** XPS survey scan spectra of V_2_AlC powder (black), laser-induced V_2_AlC powder (pink), VMX_NP_-LIG (red), and VMX_NP_-LIG_300_ (blue).


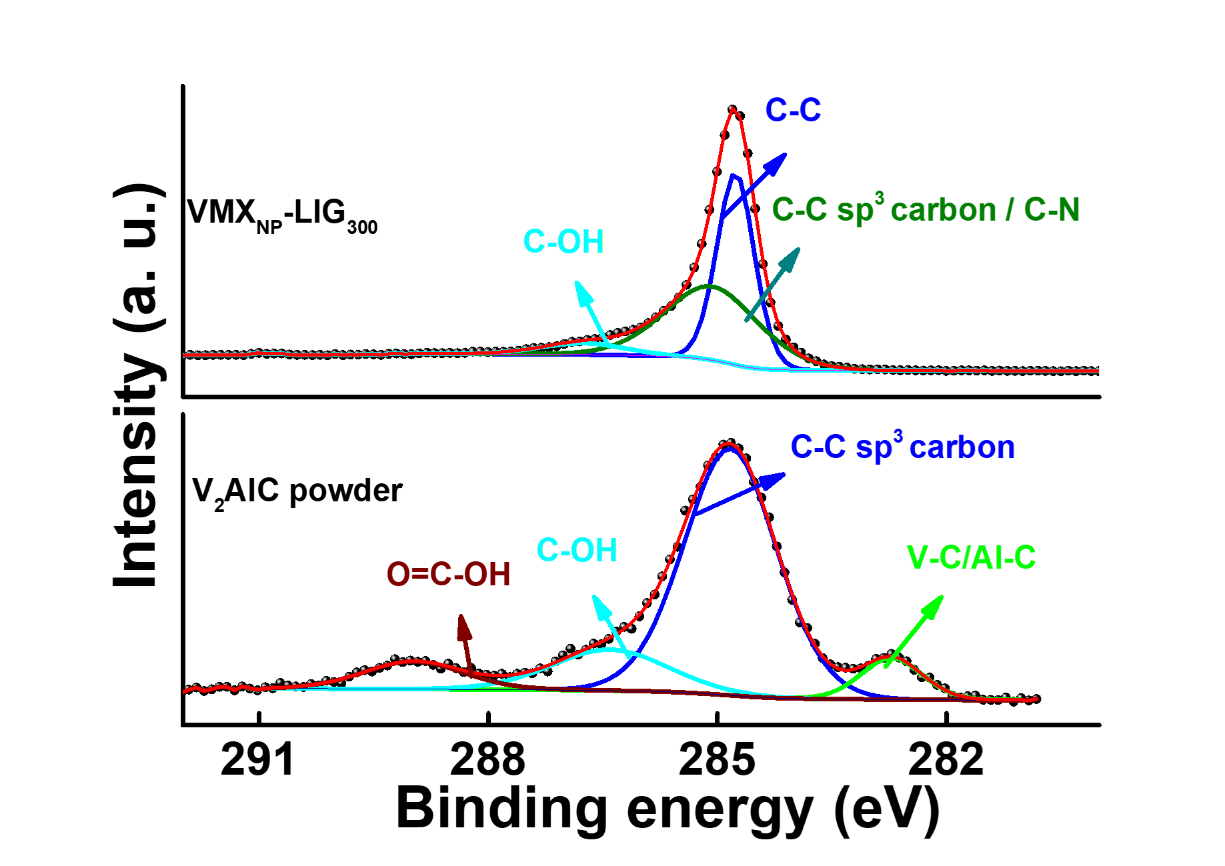


**Figure S7.** C 1s high-resolution XPS spectra of V_2_AlC powder and VMX_NP_-LIG_300_.


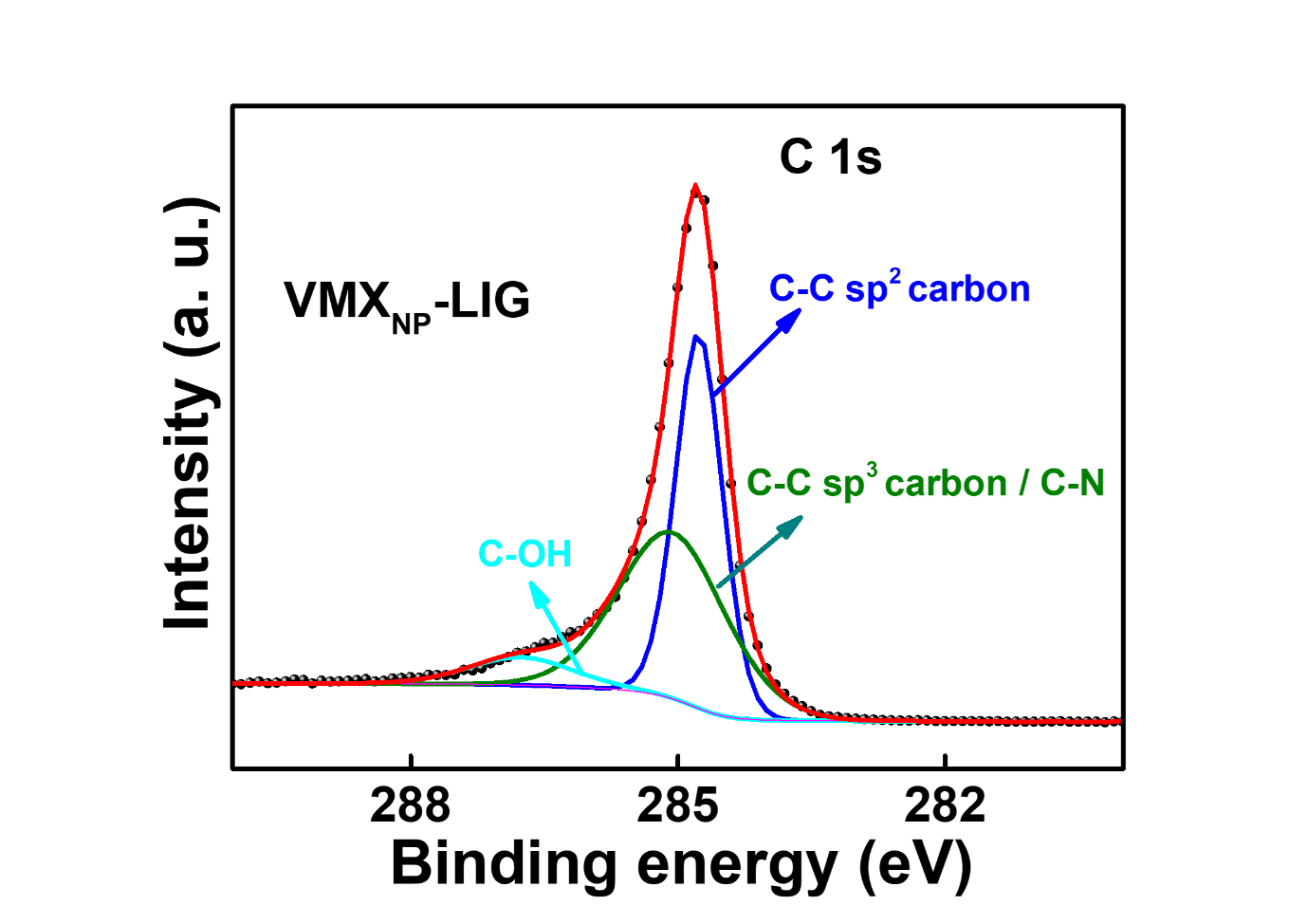


**Figure S8.** C 1s high-resolution XPS spectra of VMX_NP_-LIG.


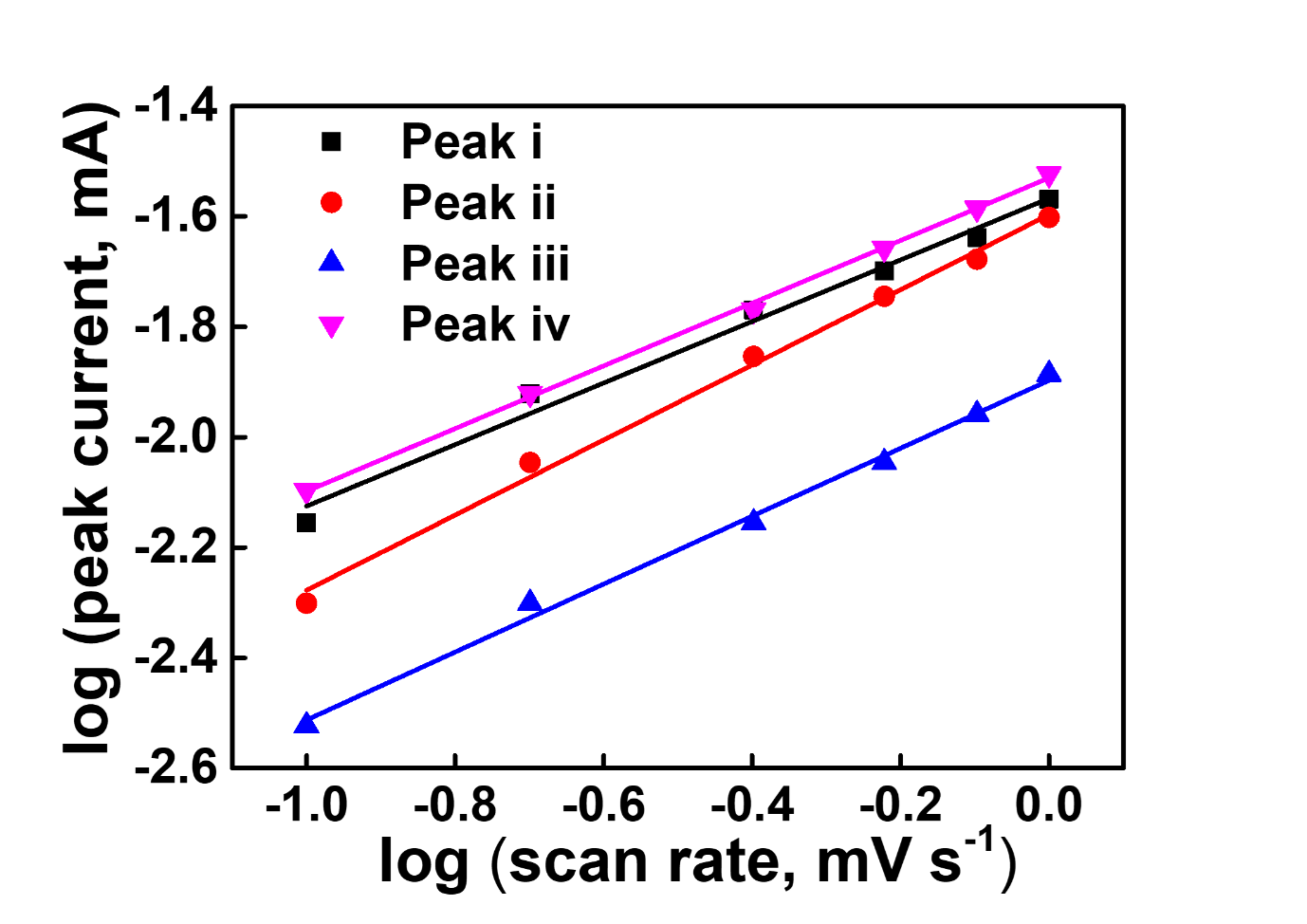


**Figure S9.** log i and log ʋ plots at specific peak currents as presented in CV curves of Figure 3b.


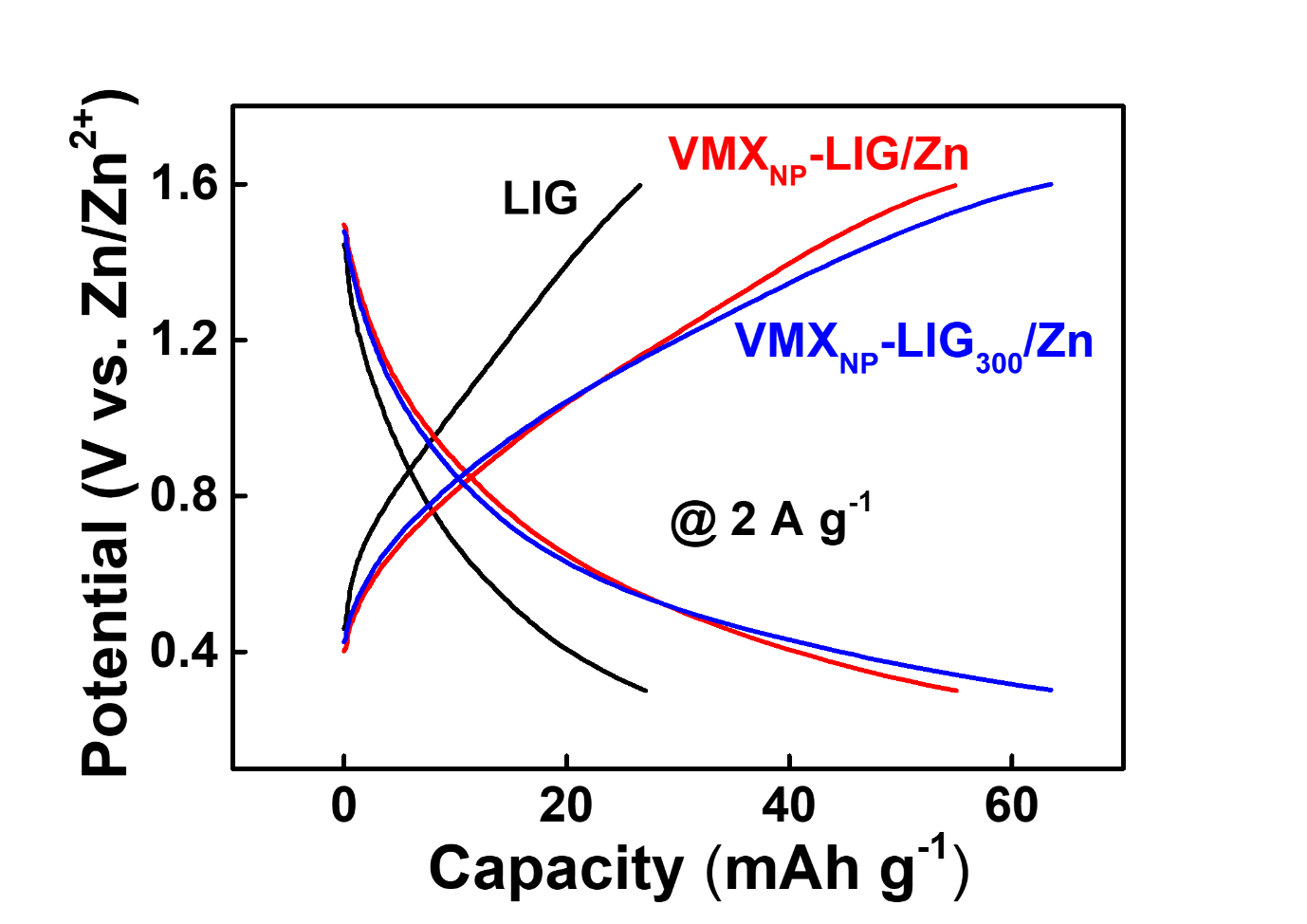


**Figure S10.** Comparative charge-discharge profile of LIG, VMX_NP_-LIG, and VMX_NP_-LIG_300_ at a current density of 2 A g^-1^.


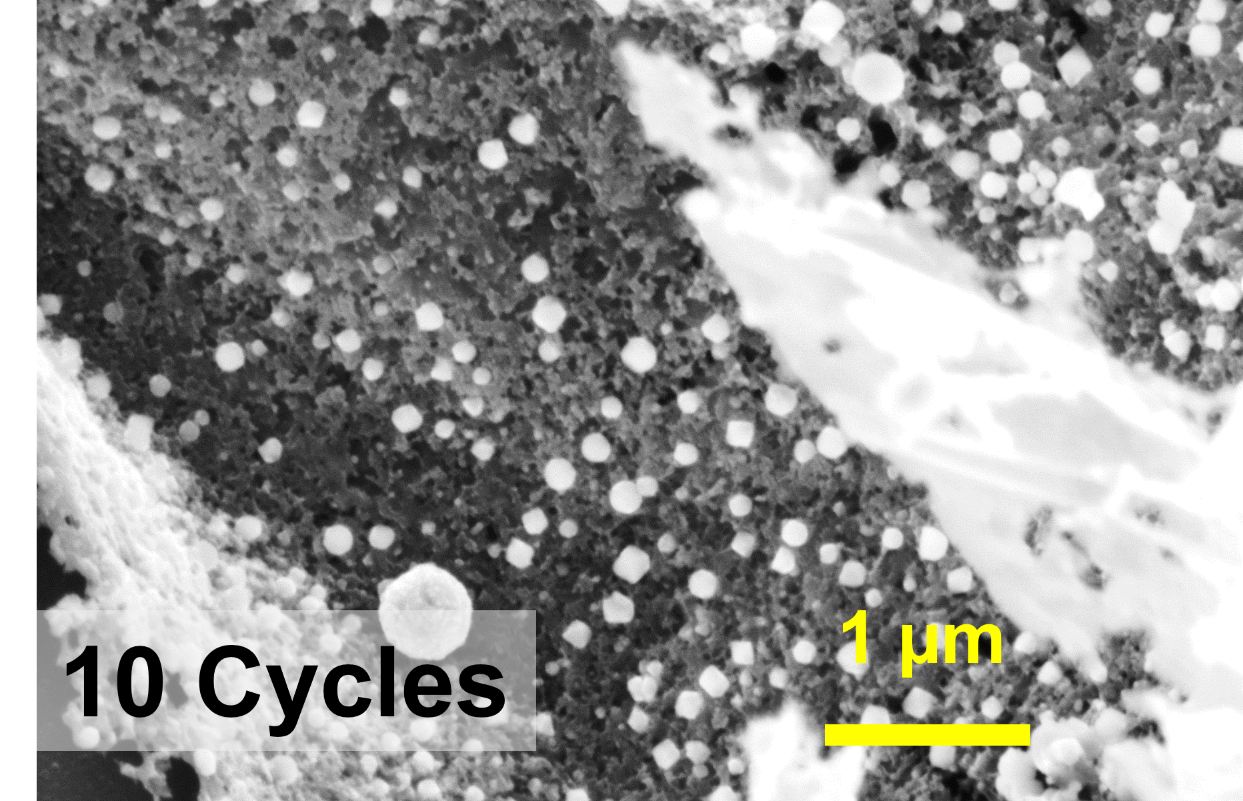


**Figure S11.** SEM images of the VMXNP-LIG_300_ cathode after 10^th^ cycles.


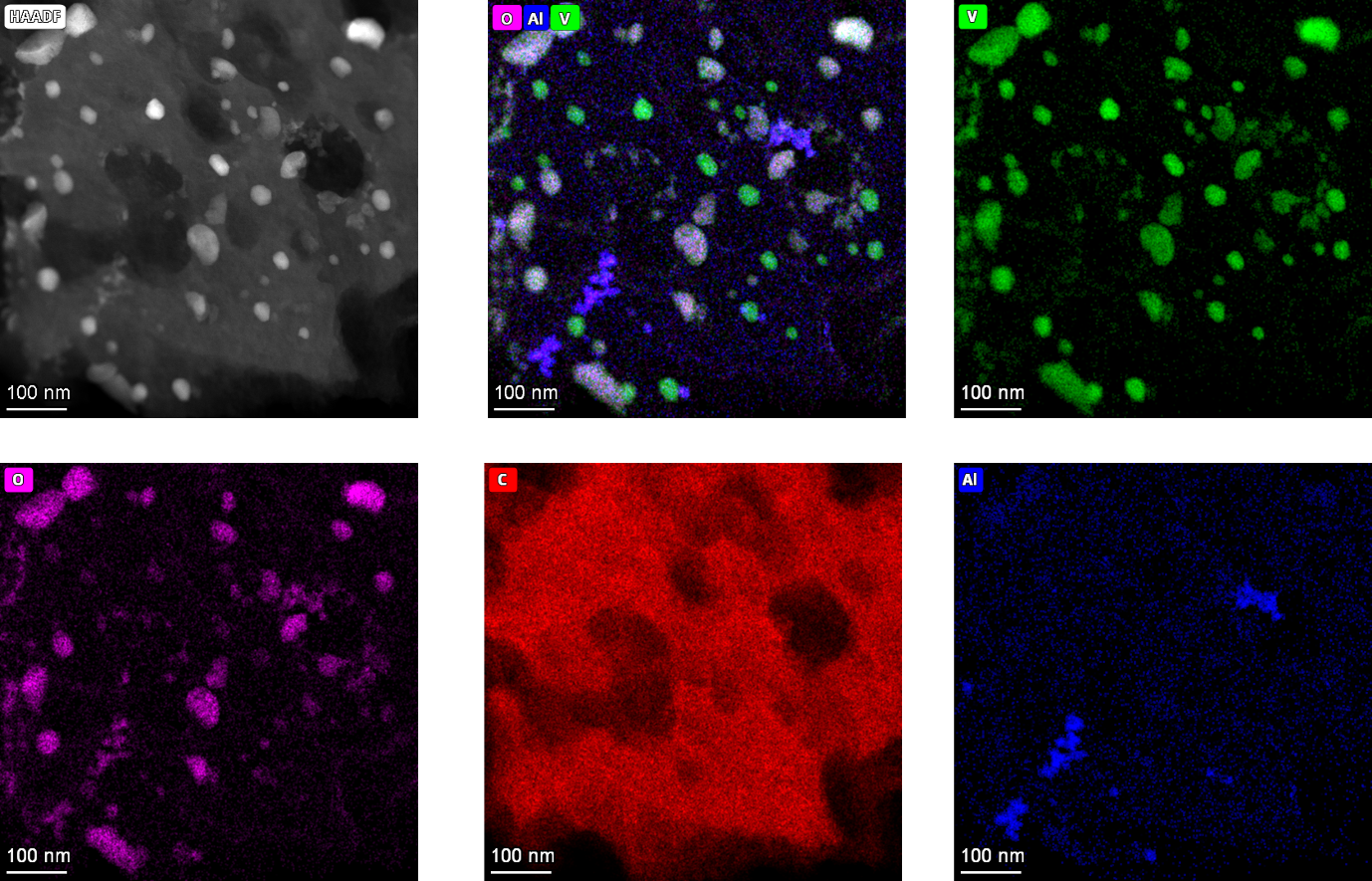


**Figure S12.** The HAADF-STEM and element mapping of V, O, C, and Al for VMX_NP_-LIG_300_ cathode after 6 k cycles.


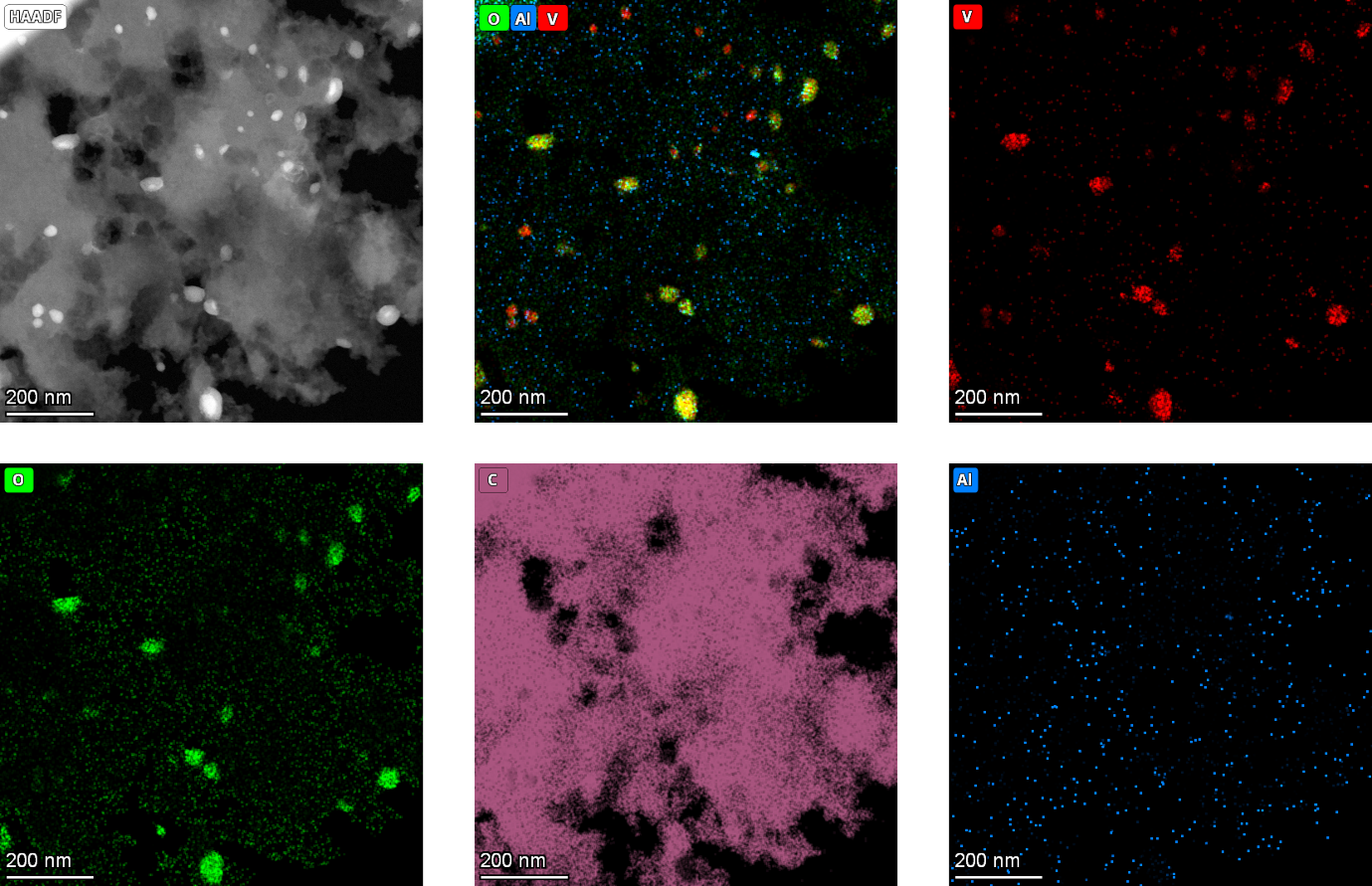


**Figure S13.** The HAADF-STEM and element mapping of V, O, C, and Al for VMX_NP_-LIG_300_ cathode after 20 k cycles.

**Figure S14.** Atom-decomposed DOS of **(a)** graphene, **(b)** V_2_C/V_2_O_5_, **(c)** gr/V_2_C/V_2_O_5_. Carbon in black, vanadium in blue, oxygen in red.

**Figure S15.** Atom-decomposed DOS of **(a)** Zn@V_2_C/V_2_O_5_, **(b)** Zn@gr/V_2_C/V_2_O_5_. Carbon in black, vanadium in blue, oxygen in red, zinc in grey.

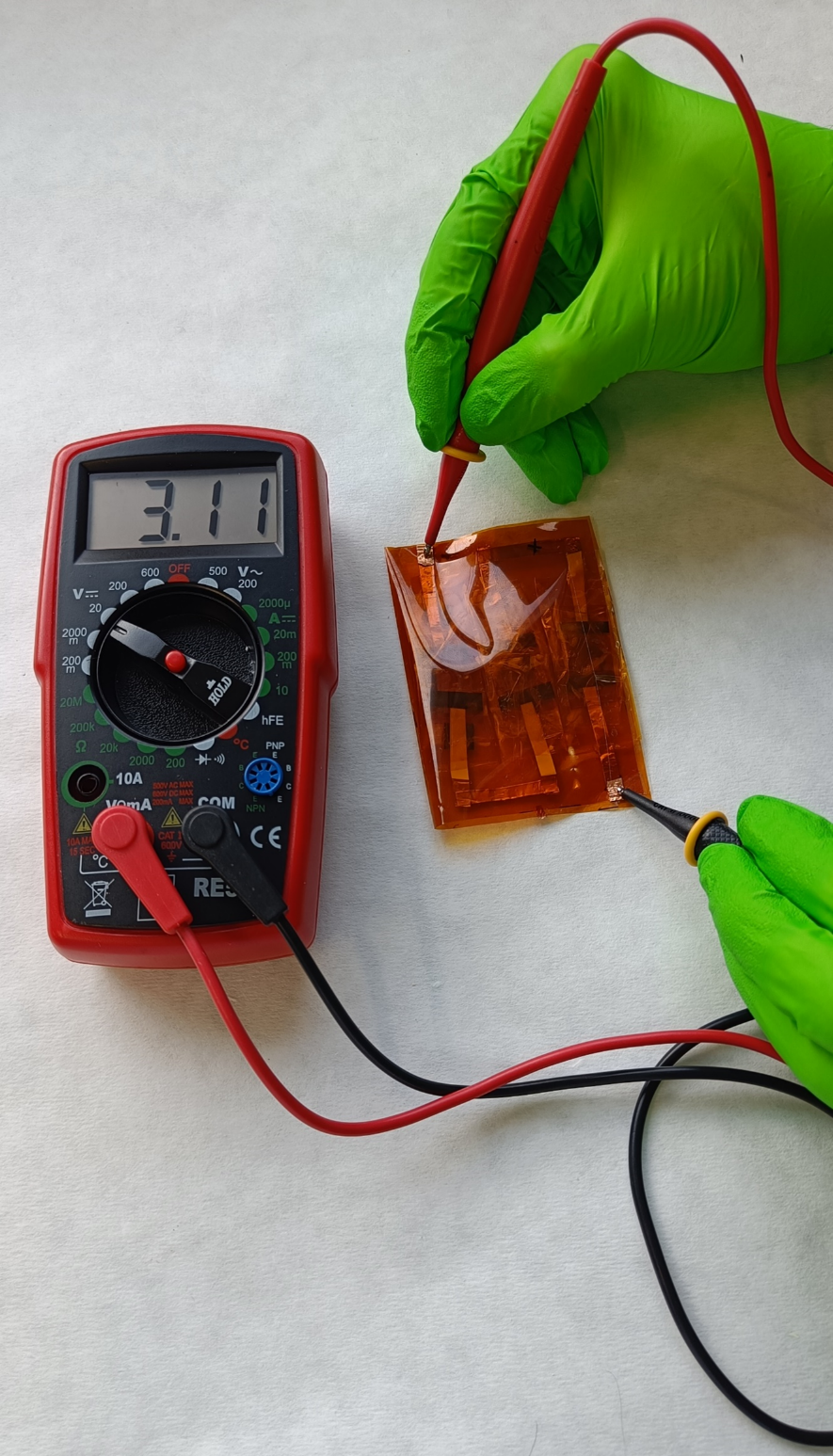


**Figure S16.** Screenshot showing the OCP value of three serially connected ZIBs.


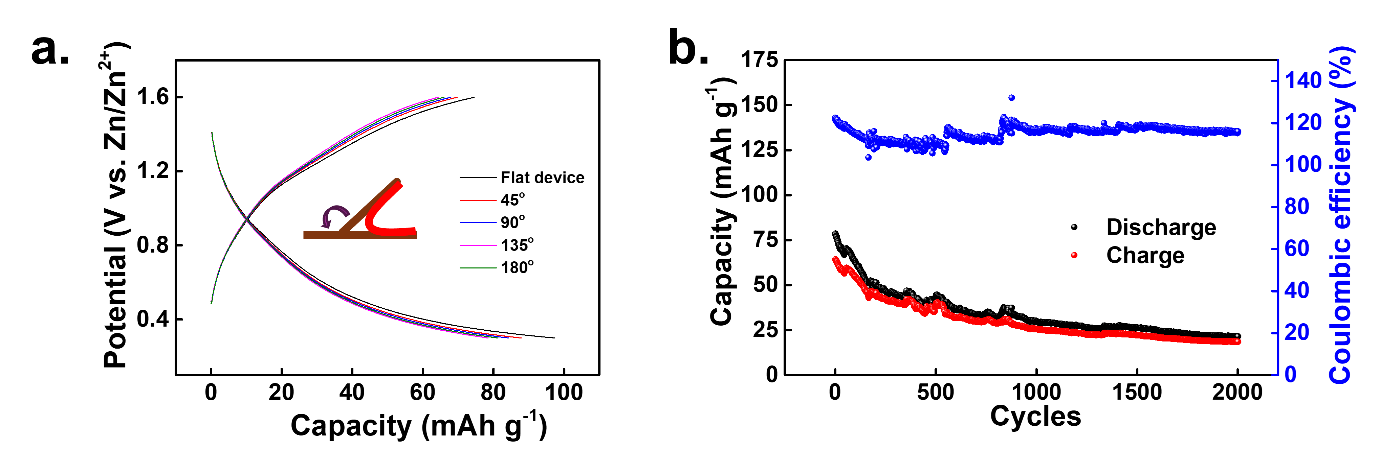


**Figure S17. (a)** Charge discharge profile of VMX_NP_-LIG_300_ under different bending conditions at a current density of 2.8 A g^-1^. (b) Cyclic stability of VMX_NP_-LIG_300_ in a fully (180^o^) bent configuration.

Table S1. Results from Raman spectra fitting.

| Sample | D band position (cm^-1^) | FWHM D band (cm^-1^) | G band position  (cm^-1^) | FWHM G band (cm^-1^) | 2D band position (cm^-1^) | FWHM 2D band (cm^-1^) | I_D_/I_G_ | I_2D_/I_G_ |
| --- | --- | --- | --- | --- | --- | --- | --- | --- |
| VMX_NP_-LIG | 1337.44 | 56.49 | 1573.24 | 52.66 | 2676.35 | 72.82 | 0.79 | 0.28 |
| VMX_NP_ -LIG_300_ | 1336.93 | 50.42 | 1569.61 | 47.70 | 2672.45 | 70.40 | 0.51 | 0.40 |

Table S2. Results from XPS survey scan.

| Sample | C 1s  At% | O 1s  At% | N 1s  At% | V 2p  At% | Al 2p  At% | V/O | Al/O | V/Al |
| --- | --- | --- | --- | --- | --- | --- | --- | --- |
| V_2_AlC | 34.42 | 39.60 | - | 5.30 | 20.67 | 0.13 | 0.52 | 0.25 |
| Laser induced  V_2_AlC | 21.12 | 49.16 | - | 5.62 | 24.10 | 0.11 | 0.49 | 0.23 |
| VMX_NP_ -LIG | 90.08 | 8.08 | 0.13 | 0.8 | 0.91 | 0.09 | 0.11 | 0.88 |
| VMX_NP_ -LIG_300_ | 87.42 | 8.68 | 0.87 | 1.64 | 1.39 | 0.18 | 0.16 | 1.20 |
